# Laser-Derived Interfacial Confinement Enables Planar Growth of 2D SnS_2_ on Graphene for High-Flux Electron/Ion Bridging in Sodium Storage

**DOI:** 10.1007/s40820-022-00829-1

**Published:** 2022-04-01

**Authors:** Xiaosa Xu, Fei Xu, Xiuhai Zhang, Changzhen Qu, Jinbo Zhang, Yuqian Qiu, Rong Zhuang, Hongqiang Wang

**Affiliations:** State Key Laboratory of Solidification Processing, Centre for Nano Energy Materials, School of Materials Science and Engineering, Northwestern Polytechnical University, Shaanxi Joint Laboratory of Graphene (NPU), Xi’an, 710072 People’s Republic of China

**Keywords:** Laser-manufacturing, Metastable, Interfacial engineering, Covalent bridging, Na-storage

## Abstract

Establishing covalent heterointerfaces with face-to-face contact is promising for advanced energy storage, while challenge remains on how to inhibit the anisotropic growth of nucleated crystals on the matrix. Herein, face-to-face covalent bridging in-between the 2D-nanosheets/graphene heterostructure is constructed by intentionally prebonding of laser-manufactured amorphous and metastable nanoparticles on graphene, where the amorphous nanoparticles were designed via the competitive oxidation of Sn-O and Sn-S bonds, and metastable feature was employed to facilitate the formation of the C-S-Sn covalent bonding in-between the heterostructure. The face-to-face bridging of ultrathin SnS_2_ nanosheets on graphene enables the heterostructure huge covalent coupling area and high loading and thus renders unimpeded electron/ion transfer pathways and indestructible electrode structure, and impressive reversible capacity and rate capability for sodium-ion batteries, which rank among the top in records of the SnS_2_-based anodes. Present work thus provides an alternative of constructing heterostructures with planar interfaces for electrochemical energy storage and even beyond.

**Supplementary Information:**

The online version contains supplementary material available at 10.1007/s40820-022-00829-1.

## Highlights

- Face-to-face covalent bridging in-between 2D-nanosheets/graphene heterostructure was constructed by intentionally prebonding of laser-manufactured amorphous and metastable nanoparticles on graphene.
- The consecutive bonding enables the robust anchoring of ultrathin SnS_2_ nanosheets on graphene with huge covalent coupling area as well as spontaneous charge transfer in-between the heterostructure.
- Such laser-manufactured heterostructure is capable of guaranteeing high-flux electron/ion migration and structural integrity upon cycling, thus contributing to the unprecedented Na-storage capability.

**Supplementary Information:**

The online version contains supplementary material available at 10.1007/s40820-022-00829-1.

## Graphical abstract

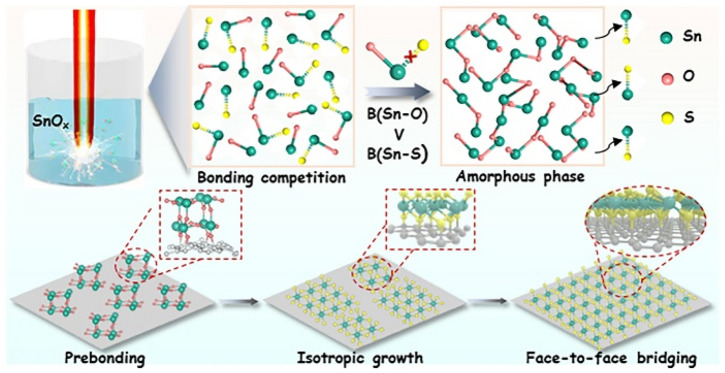

## Introduction

Given the high theoretical capacity and superior structural stability, hybrids of nano-active material anchored on conductive skeletons have recently witnessed significant success as promising candidates for electrochemical ions storage [1–6]. The as-acquired composites demonstrate encouraging potentials for metal ions storage, while the manners of interfacial interaction between nano-active material and conductive matrix have been recognized as the dominate factor of restricting the energy storage performance [1, 7–13]. The poor interfacial coupling would result in sluggish electron/ion transfer kinetics and inevitable aggregation of active materials, giving rise to rapid capacity decay and inferior rate capability [11]. Preliminary successes have been achieved via interfacial regulations such as Van der Waals' force, electrostatic interaction and chemical coordination to enhance interfacial coupling in-between heterostructures [10–22]. Among them, construction of covalent bridging has been demonstrated to maximize the reinforcement of ions-storage performance, rising from that the covalent bridging can directly boost charge-transfer kinetics by functioning as electron/ion transfer channel between nano-active material and conductive matrix [1, 7–10]. Developing rational interfacial engineering strategies to construct robust covalent bridging in-between heterostructures has thus been of paramount significance for pursuing highly efficient energy storage.

The recent explorations have been devoted to the construction of reinforced interface covalent bridging for highly efficient electrochemical ions storage. In fact, most active materials tend to be anchored on conductive skeleton in a manner of discrete nanoparticles (NPs) embedding [23, 24]. For example, employment of coordination interaction results in a sequence of heterostructures of NPs anchored on carbon materials with M-C or M-X-C (M=metal atoms; X=O, S, N etc.) bonds, and the enhanced interfacial bridging by covalent bonds is deemed as the role of accelerating the ions-storage capability [7, 10–13]. Nevertheless, the electrochemical performances of the heterostructures are still unsatisfied because the formed point-to-face contact is incapable of supporting high-flux interfacial electron/ion bridging. Investigation has thus been undertaken on the construction of 2D heterostructures with high-capacity nanosheets anchored on carbon skeleton [25, 26]. A notable example is that an enhanced Na-storage capacity of 88 mAh g^−1^ at 20.0 A g^−1^ was achieved with well-designed 2D SnSe nanoplates anchoring on N-doped carbon as anode [27]. This spurs the potential endeavors on exploring high-flux electron/ion transfer kinetics by vividly modulating the covalent interfacial bridging for boosted electrochemical energy storage. A challenge remains however on how to kinetically inhibit the vertical growth of 2D nanostructures on carbon matrix in cases of using metal salt-based precursors, which leads usually to the line-to-face contact in interfacial bridging, instead of the expected face-to-face contact that more favors covalent bridging with high-flux interface coupling.

**Fig. 1:**
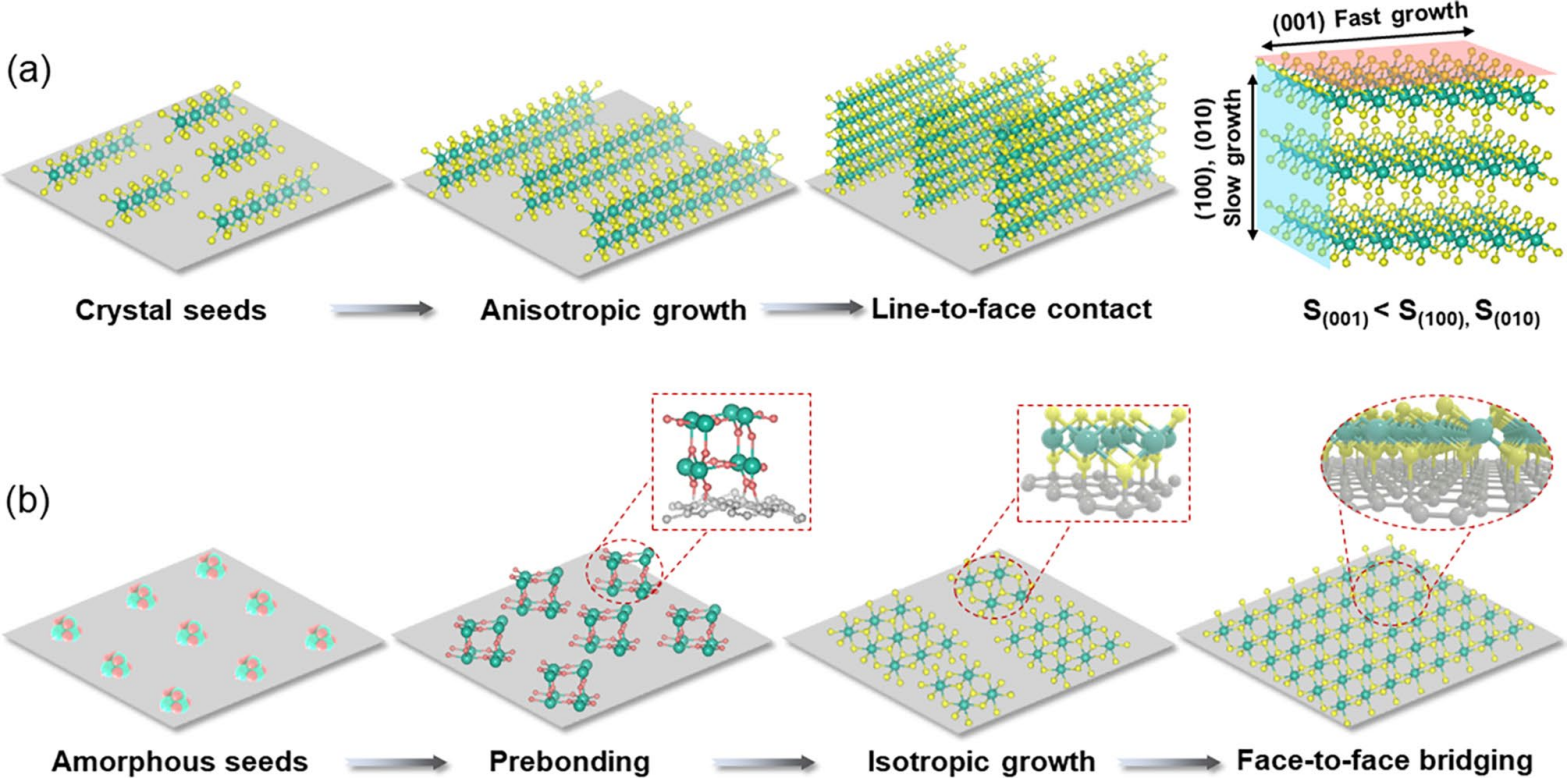
Schematic illustration of the interfacial confinement for synthesizing 2D heterostructures with face-to-face contact. **a** Line-to-face contact in case of using crystal seeds (S: Surface energy). **b** Face-to-face contact in case of using amorphous seeds

Herein, we demonstrate an efficient strategy of accessing face-to-face covalent bridging of 2D nanosheets with matrix of graphene for high-flux electron/ion transfer, by taking advantage of the prebonding of laser-manufactured metastable NPs on graphene, as well as the amorphous feature of the nucleated NPs that drives the parallelly epitaxial growth of the anchored 2D nanosheets. As depicted in Fig. 1a, the seed crystals nucleated on the surface of carbon matrix usually undergo anisotropic growth, due to the large surface energy differences between diverse crystal planes [28–30]. Consequently, crystal nuclei of 2D materials favor to grow along the (100) and (010) planes with higher surface energy [31, 32], forming nanostructures of 2D nanosheets standing on carbon matrix, i.e., heterostructures with line-to-face contact. On the contrary, by interfacial confining the anisotropic growth of the crystal nuclei via making the nuclei amorphous, as depicted in Fig. 1b, the nuclei anchored on the matrix have the tendency of growing along the (001) plane with low surface energy, owing to isotropic growth behavior of the nucleates [28, 33]. Such inhibition of the anisotropic growth would thus allow for transfer of the line-to-face growth of 2D nanosheets, to the face-to-face covering of 2D nanosheets on graphene. Therefore, under the synergism of isotropic growth derived by amorphous state and robust prebonding, the growth of 2D nanosheets is expected to interfacial confined to undergo the structural reorganization and epitaxial growth parallelly to the conductive matrix.

By following the above scenario, we successfully achieve the synthesis of SnS_2_/graphene heterostructure (A-SnS_2_@G) with face-to-face contact and robust interfacial C-S-Sn bonds, through hydrothermal treatment of laser-manufactured amorphous supranano SnO_*x*_ (~ 4 nm) and graphene oxide (GO) in the presence of thiourea. Benefiting from the continuous and high-flux interfacial bridging and ultrathin dimension of SnS_2_ (~ 4 layers), which can render unimpeded electron/ion transfer pathways, more ion-storage sites and improved structural stability, the delicate-designed A-SnS_2_@G heterostructure delivers much higher Na-storage performance compared to those of SnS_2_/G composite and pure SnS_2_ nanosheets. In detail, cycling stability of 597 mAh g^−1^ at 0.2 A g^−1^ after 200 cycles and unprecedented rate capability of 259 mAh g^−1^ at 20.0 A g^−1^ can be obtained, which is among the top in the records of all the SnS_2_-based anodes for Na-ion batteries (SIBs). The Na-storage mechanism and structural evolution of A-SnS_2_@G electrode were elucidated by various in/ex situ characterizations as well as electrochemical kinetic analysis. Theoretically, systematic density functional theory (DFT) simulations further demonstrate the significance of consecutive C-S-Sn bonds to enhance the charge transfer and intimate adhesion of SnS_2_ and graphene matrix. This work not only provides an efficient strategy of constructing heterostructures with face-to-face contact for high-performance ion-storage electrodes, but also contributes to an in-depth understanding of interfacial covalent bridging for the enhancement of electrochemical behavior.

## Experimental Section

### Synthesis of Metastable A-SnO_***x***_ NPs

The technique of pulsed laser manufacturing in liquid was employed for the synthesis of A-SnO_*x*_ NPs, and a pulsed Nd:YAG laser (1064 nm, 10 Hz, 10 ns) was used as the light source. Before the laser ablation, the Sn target (99.99% purity) was polished and washed with ethanol for several times to remove the surface oxide layer. Then the target was immersed in 7 mL thiourea aqueous solution (20 mg mL^−1^) and ablated for 1 min under a continuous ultrasonication to get the colloidal solution containing amorphous SnO_*x*_. The optimal laser fluence was 800 mJ pulse^−1^ cm^−2^.

### Synthesis of A-SnS_2_@G Heterostructure

GO was synthesized via the modified Hummers method. Firstly, the suspension of 50 mg GO in 50 mL deionized water was ultrasonication for 1 h to obtain the GO aqueous solution. Then 200 mL laser-manufactured thiourea aqueous solution consisting of A-SnO_*x*_ was dropped into the GO aqueous solution slowly under drastic magnetic stirring to ensure uniform mixing. After that, the mixture was transferred to Teflon-lined stainless-steel autoclave and heated at 200 °C for 12 h. The resultant A-SnS_2_@G was obtained by centrifugated and freeze-drying after cooling to room temperature. The pure SnS_2_ sheets were synthesized under the same conditions but without GO.

### Materials Characterization

The morphology and microstructure of the as-obtained materials were characterized by transmission electron microscopy (TEM, Talos F200X, FEI) and scanning electron microscopy (SEM, NANOSEM450, FEI). The Zeta potential was tested by dynamic light scattering (Malvern Zetasizer Nanoseries) at room temperature. The crystal phase was determined by X-ray diffractometer (XRD, Bruker D8 ADVANCE) with Cu Kα radiation (*λ* = 1 0.15406 nm). In situ Raman spectra were collected on a Renishaw inVia Raman Microscope (532 nm) combined with CV test at 1.0 mV s^−1^. The surface chemistry state of the samples was explored by X-ray photoelectron spectroscopy (XPS, Shimadzu Kratos Axis Supra). Fourier transform infrared (FTIR) spectra were measured via a Thermo Scientific Nicolet iS5 (wavenumber range 4000–400 cm^−1^). N_2_ adsorption/desorption isotherms were monitored on a Micromeritics ASAP 2020 analyzer to determine the Brunauer–Emmett–Teller (BET) surface area. DFT was performed to analyze the pore size distributions. The loading of SnS_2_ was detected via a TG/DTA analyzer (METTLER TOLEDO) in air atmosphere.

### Electrochemical Measurements

The working electrodes were prepared by casting slurries consisting of active material (80 wt%), Super P (10 wt%), carboxymethyl cellulose (CMC, 8 wt%) and styrene-butadiene rubber (SBR, 2 wt%) in distilled water onto Cu foil and then dried in a vacuum furnace at 80 °C for 12 h. The average mass loading of each electrode was ~ 1.0 mg cm^−2^. The coin cells (CR2032) were assembled in an Ar-filled glovebox (H_2_O and O_2_ < 0.1 ppm) with metallic sodium foil as the counter electrode and GF/D glass fiber (Whatman) as the separator. The electrolyte was 1 M NaClO_4_ dissolved in 1:1 volume ratio of ethylene carbonate (EC) and dimethyl carbonate (DMC) with 5% fluoroethylene carbonate (FEC), and the volume of electrolyte for each coin cell was ~ 80 μL. Galvanostatic charge/discharge tests were carried out using a LAND-CT2001A multichannel galvanostat within the voltage window of 0.01–3.0 V. The specific capacity contribution was based on the mass of A-SnS_2_@G or SnS_2_. A CHI 660E electrochemical workstation (Shanghai Chenhua) was used to record cyclic voltammogram (CV) and electrochemical impedance spectroscopy (EIS, 100 kHz-0.01 Hz) curves. For full-cell assembly, the cathode was a mixture of Na_3_V_2_(PO_4_)_3_, Super P and PVDF in the weight ratio of 80:10:10. The capacity ratio of cathode/anode was ~ 1.2:1 in a typical full cell. Galvanostatic charge/discharge was conducted between 0.6 and 3.8 V, and the specific capacity was calculated based on the mass of anode.

## Results and Discussion

### Synthesis and Characterization of Materials

**Fig. 2:**
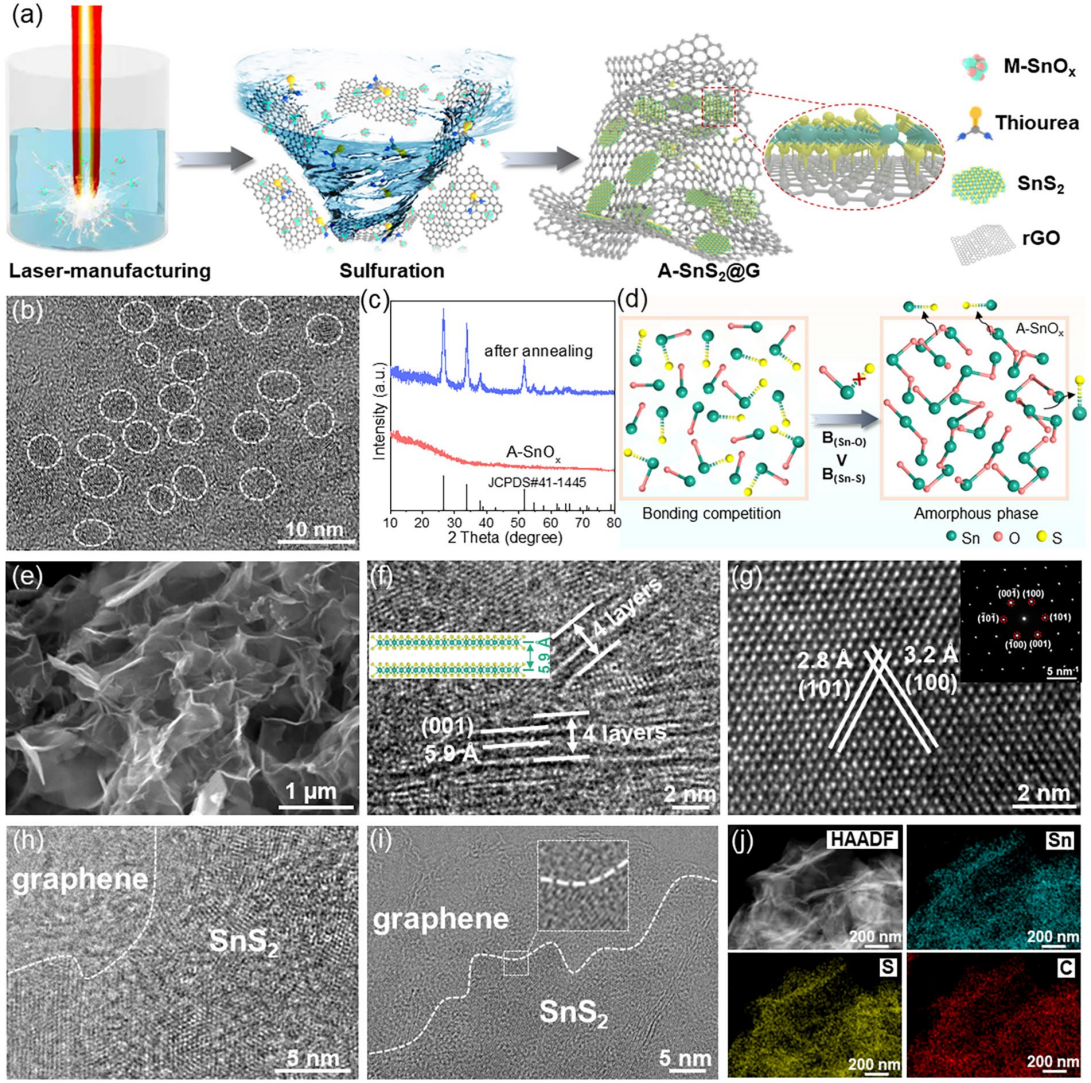
**a** Construction of A-SnS_2_@G heterostructure. **b** TEM image of A-SnO_*x*_. **c** XRD spectra of A-SnO_*x*_ and annealing in nitrogen. **d** Schematic illustration of synthesis of A-SnO_*x*_ by bonding competition (B: bonding energy). **e** SEM image and **f**–**i** HRTEM images of A-SnS_2_@G (the inset in Fig. 2**g** is corresponding SAED pattern). **j** Elemental mapping images of A-SnS_2_@G

Experimentally, we achieve the synthesis of amorphous tin oxides (A-SnO_*x*_) for pre-bonding by a modified procedure of laser manufacturing in liquid. It was reported in our previous work that crystalline SnO_*x*_ could be generated from the irradiation of a non-focused pulsed laser beam (1064 nm, 10 Hz, 10 ns) [8], while in present study we placed polished Sn foil in thiourea aqueous solution upon laser irradiation. As schematically illustrated in Fig. 2a, Sn vapor evaporated by high-energy laser beam undergoes incomplete oxidation in low-temperature thiourea aqueous solution (~ 0 °C), in which both H_2_O and H_2_S (produced by thiourea decomposition) are expected to participate in the oxidation of Sn vapor. In detail, local ultra-high temperature and ultra-high pressure Sn plasma with high reactivity is produced instantaneously via the violent interaction between high-energy laser beam and Sn foil in thiourea aqueous solution. Momentarily, both H_2_O and H_2_S in thiourea aqueous solution participate in the oxidation of Sn plasma. Accompanied by the rapid temperature quenching, Sn plasma is not oxidized thoroughly, thus producing abundant metastable and amorphous A-SnO_*x*_. TEM image shows that the laser-manufactured NPs present uniform size of ~ 4 nm and no lattice fringes can be observed (Fig. 2b), indicating the amorphous feature of the as-obtained supranano particles, which is further confirmed by XRD pattern (Fig. 2c). However, after annealing the sample in nitrogen, the typical peaks in XRD and Raman patterns could be identified to SnO_2_, indicating that the laser-manufactured NPs are amorphous SnO_*x*_ (Figs. 2c and S1). Different from the case of laser irradiation of Sn in water [8], the irradiation of Sn in thiourea aqueous solution allows for oxidization of Sn upon both H_2_O and H_2_S, where both Sn-O and Sn-S bonds could form in the oxidation of Sn vapor. However, the generated Sn-S bond was eventually replaced by Sn-O bond owing to its lower binding energy (467 kJ mol^−1^) than that of Sn-O (528 kJ mol^−1^). The formation of the amorphous supranano SnO_*x*_ can thus be ascribed to the competitive bonding between Sn-O and Sn-S, which would deepen the crystallization disorder of laser-manufactured particles, as depicted in Fig. 2d, thus differing from the case of generating crystalline SnO_*x*_ NPs in aqueous solution [8].

In contrast to the white color of SnO_2_, the dark brown of laser-manufactured A-SnO_*x*_ suggests the presence of plenty of oxygen vacancies (Fig. S2) [34, 35], which was confirmed by the analysis of X-ray photoelectron spectroscopy (XPS). As shown in Fig. S3c, the Sn 3*d*_5/2_ and 3*d*_3/2_ peaks of A-SnO_*x*_ show obvious downshift compared with the tin (IV) in SnO_2_, manifesting the existence of abundant oxygen vacancies [36, 37]. The three fitted peaks presented in O 1* s* spectrum of A-SnO_*x*_ assign to the lattice oxygen (O_L_), oxygen vacancies (O_V_) and adsorbed oxygen (O_A_), respectively (Fig. S3d) [35, 37]. It should be noted that the obtained A-SnO_*x*_ NPs are expected to prebond easily on reduced graphene oxide (rGO) with covalent bonds, benefitting from the extremely high reactivity enabled by the amorphous phase and abundant oxygen vacancies, as demonstrated in our recent experiment and theoretical simulation [8]. Moreover, the generated A-SnO_*x*_ colloids have positively charged surface (+ 11.7 mV) (Fig. S4), which favors for the uniform and intimate adsorption on the oppositely charged (− 32.3 mV) GO surface by electrostatic attraction and chelation. Therefore, both the metastable feature and the positively charged surface of A-SnO_*x*_ facilitate the prebonding of laser-generated supranano particles on the matrix of negatively charged GO. We therefore transfer the as-obtained colloidal solution into autoclave for subsequent hydrothermal treatment, to achieve the synthesis of heterostructure of 2D nanosheets on graphene, with the expected face-to-face covalent bonding.

**Fig. 3:**
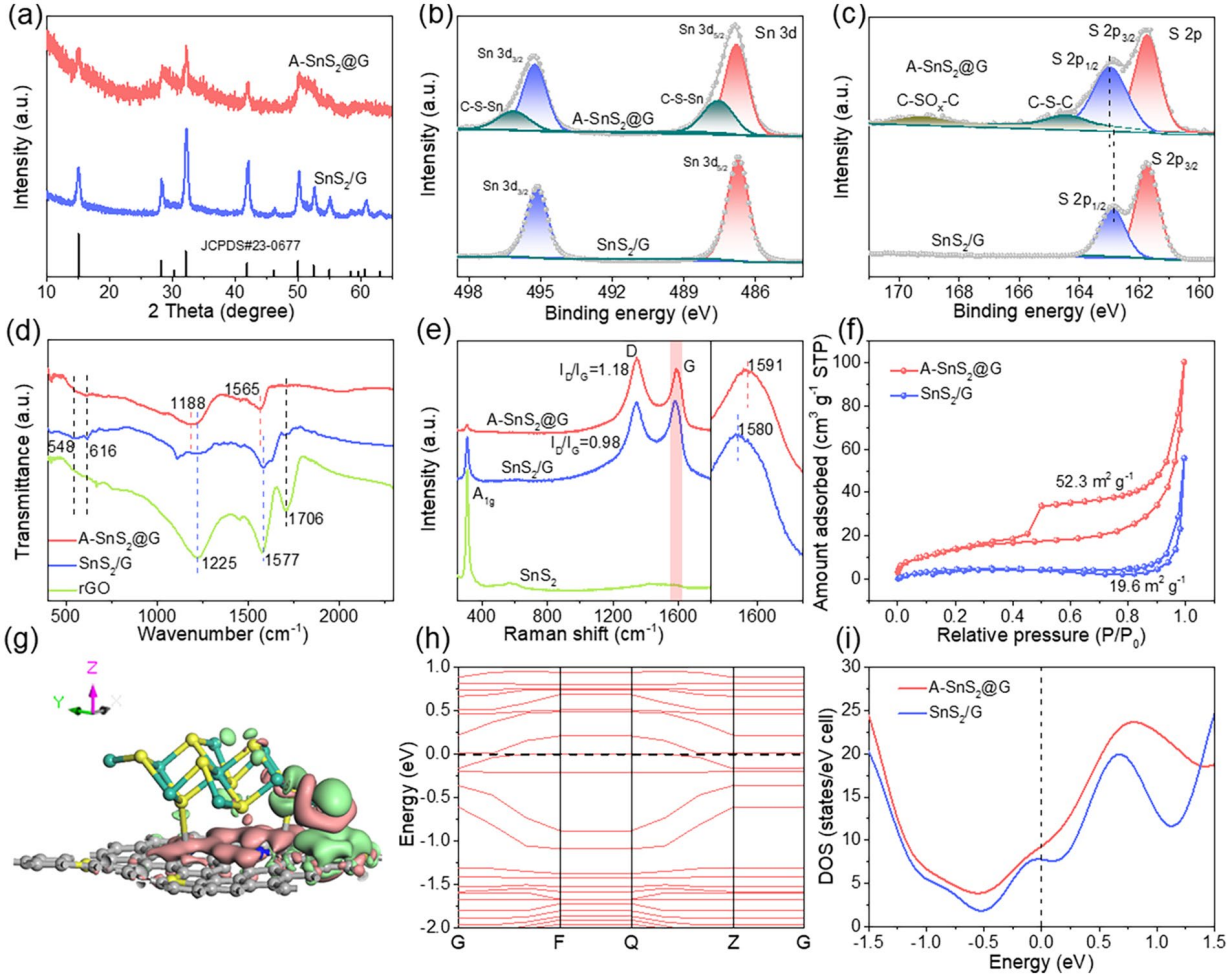
**a** XRD, **b** XPS Sn 3*d* and **c** S 2*p* spectra of A-SnS_2_@G and SnS_2_/G. **d** FTIR spectra of A-SnS_2_@G, SnS_2_/G and rGO. **e** Raman patterns of A-SnS_2_@G, SnS_2_/G and SnS_2_. **f** Nitrogen adsorption–desorption isotherms of A-SnS_2_@G and SnS_2_/G. **g** Charge density difference and **h** band structure of optimized A-SnS_2_@G model (the green/pink cloud represents the accumulation/depletion of electrons). **i** Calculated DOS of A-SnS_2_@G and SnS_2_/G around the Fermi level

As shown in Fig. 3a, XRD pattern of the product demonstrates all the apparent peaks identified well to SnS_2_ (JCPDS No. 23-0677), where the broadening with decreased intensity of the characteristic peaks indicates the obtained A-SnS_2_@G might have few-layered structures. The SEM and TEM images show that the as-prepared A-SnS_2_@G heterostructure displays a fluffy three-dimensional wrinkle-like structure, in which SnS_2_ with few-layers structure (~ 4 layers) is intimately anchored on graphene without agglomeration (Fig. 2e, f). The interlayer spacing measured to be 5.9 Å, assigning to the (001) plane of hexagonal SnS_2_. The distinct crystalline lattices of SnS_2_ can be clearly observed in the HRTEM image, and the measured interplanar distances are 3.2 and 2.8 Å, corresponding to the (100) and (101) planes, respectively (Fig. 2g). The sharp diffraction spots shown in the selected area electron diffraction (SAED) pattern (inserted in Fig. 2g) further indicate the as-synthesized SnS_2_. With the synergism of isotropic growth and robust interface confinement induced by prebonding of amorphous A-SnO_*x*_ seeds, the vertical growth was inhibited and parallelly epitaxial growth was well driven. The grown ultrathin SnS_2_ nanosheets exposed to (001) plane are intimately adhered to the graphene matrix with face-to-face contact (Fig. 2h, i), which is significantly different from the island growth with line-to-face contact presented in the previous literatures [9, 27, 38–41]. Such optimized face-to-face contact is expected to render successive heterointerface for huge covalent bridging area and high-flux electron/ion transfer.

The energy-dispersive X-ray spectroscopy (EDS) mappings (Fig. 2j) reveal that the expected elements (Sn, S, C) are homogeneously distributed throughout the A-SnS_2_@G. According to the thermogravimetric analysis, the SnS_2_ content in A-SnS_2_@G is calculated to be 87.2% by assuming the remaining phase in air atmosphere is SnO_2_ (Fig. S5). Herein, it should be noted that under the pulsed laser irradiation in thiourea aqueous solution, the incomplete decomposition of thiourea leads to insufficient sulfur vapor pressure and the reaction time is also transient (10 ns pulse^−1^), which leads to the generation of SnO_*x*_ NPs. Different from the LML technique [42–46], hydrothermal in thiourea aqueous solution could enable long-time vulcanization in ample sulfur source from the complete decomposition of thiourea under high temperature and pressure, and A-SnO_*x*_ can undergo adequate structural reorganization for the growth of SnS_2_ nanosheets. SnS_2_ sheets (~ 24 layers) well covering on graphene were also achieved with large laser-manufactured SnO_*x*_ (~ 25 nm) particles as precursor (Fig. S6), implying that supranano dimension (~ 4 nm) is important for the formation of ultrathin stacking. As a control, the pure SnS_2_ nanosheets (Fig. S7) were prepared by hydrothermal sulfuration of A-SnO_*x*_ without GO. Also, the thick SnS_2_ sheets (~ 35 layers) with similar content of 88.4% are randomly aggregated on rGO by direct hydrothermal of as-prepared pure SnS_2_ sheets and GO (named as SnS_2_/G, Figs. 3a and S8), indicating the formation of the heterostructure of 2D nanosheets on carbon matrix with face-to-face contact is strongly dependent on the laser-manufactured amorphous A-SnO_*x*_ with oxygen vacancies.

XPS was performed to investigate the composition and bonding state of A-SnS_2_@G and SnS_2_/G. The XPS survey spectra reveal the existence of Sn, S, C and O elements in A-SnS_2_@G and SnS_2_/G (Fig. S9a, b). Compared with SnS_2_/G, besides the fitted Sn 3*d*_5/2_ (486.8 eV) and 3*d*_3/2_ (495.3 eV) peaks [47], another pair of satellite peaks located at 487.5 and 496.2 eV appears in A-SnS_2_@G (Fig. 3b), which indicates the formation of C-S-Sn bonds, similar to those with the presence of C-S-M (e.g., M is Mo) [41]. The S 2*p* spectrum of SnS_2_/G displays two peaks at 161.8 (S 2*p*_3/2_) and 162.9 eV (S 2*p*_1/2_), corresponding to the S^2−^ of SnS_2_. In contrast, the S 2*p*_1/2_ shifted to higher binding energies in A-SnS_2_@G, indicating the robust interface coupling between SnS_2_ and graphene (Fig. 3c). Furthermore, another apparent peak at 164.4 eV can be assigned to the C-S-C bond and the weak peak at 169.3 eV is from the C-SO_*x*_-C sulfone bridge [15, 41, 47–49]. Moreover, the C-S bonds can also be detected by the peaks at 285.5 eV in the C 1*s* spectrum (Fig. S9c). The covalent bridging was further corroborated by Fourier transform infrared (FTIR) spectra (Fig. 3d). The adsorption peaks at 548 and 616 cm^−1^ are attributed to Sn-S bond [50]. The peaks at 1225, 1577 and 1706 cm^−1^ are assignable to C-O vibration of the carboxy, C=C stretching vibration of the benzene ring and C=O stretching vibration of the carboxy, respectively. Compared with SnS_2_/G and rGO, the adsorption peaks corresponding to the C-O and C=C bonds in A-SnS_2_@G downshift to 1188 and 1565 cm^−1^. It can also be observed that the C=O bond disappears, implying the robust C-S-Sn coupling effect between SnS_2_ and rGO [8, 17]. Hence, a strong interfacial interaction with face-to-face contact was constructed in the A-SnS_2_@G.

The Raman spectra were conducted to further explore the structure of as-prepared samples (Fig. 3e). Compared with SnS_2_/G and pure SnS_2_, the obvious weakness of the peak at 310 cm^−1^ (A_1g_ mode for SnS_2_) in A-SnS_2_@G implies that ultrathin SnS_2_ nanosheets were tightly wrapped with graphene. The intensity ratio of *I*_*D*_ (disordered activated D-band) to *I*_*G*_ (in-plane vibrational G-band) (*I*_*D*_*/I*_*G*_) for A-SnS_2_@G (~ 1.18) is higher than that of SnS_2_/rGO (0.98), revealing that the covalent anchoring of ultrathin SnS_2_ nanosheets increases the disorderliness and inhibits the stacking of graphene layers [15]. Intriguingly, the G-band of A-SnS_2_@G upshifts to 1591 cm^−1^ in comparison with the SnS_2_/G (1580 cm^−1^), further demonstrating the strong charge transfers in-between A-SnS_2_@G heterostructure [17]. From Fig. 3f, the BET surface area of A-SnS_2_@G (52.3 m^2^ g^−1^) is higher than that of SnS_2_/G (19.6 m^2^ g^−1^), highlighting that the ultrathin SnS_2_ nanosheets with covalently anchoring on graphene stretch the graphene layers as a spacer. Pore size distribution (Fig. S10) curves and detailed pore parameters (Table S1) obtained via the DFT reveal a hierarchical meso/macro-porosity and more mesoporous exist in A-SnS_2_@G, consisting with both the SEM and TEM analysis.

DFT calculations were further conducted to reveal qualitatively the modified electron transfer between SnS_2_ and the matrix upon forming covalent bridging. The model systems were optimized based on the (2 × 2) SnS_2_ monolayer on (6 × 6) graphene matrix (GRAPHENE or G). As known, the charge density changes (Δ*ρ*) can be acquired by Eq. (1):

1 $$\Delta \rho = \rho_{{{\text{A}} - {\text{SnS}}_{2} {\text{@G}}}} - \rho_{{{\text{SnS}}_{2} }} - \rho_{{{\text{graphene}}}}$$

where *ρ*_A−SnS2@G_, *ρ*_SnS2_ and *ρ*_graphene_ mean the charge densities of A-SnS_2_@G, SnS_2_ and graphene, respectively [14]. Thanks to the robust covalent bridging in-between SnS_2_ and graphene by C-S-Sn bonds, massive charge transfer from graphene to SnS_2_ occurs spontaneously at the interfacial region of A-SnS_2_@G (Fig. 3g), highlighting the negative polarization of the interface and unimpeded electron transfer pathways between SnS_2_ and graphene. In contrast, the rare charge density in the interface of SnS_2_ and graphene implies its weak interaction and poor charge transfer (Fig. S11a). Semi-quantitatively, the quantity of charge transfer for A-SnS_2_@G (0.18 e) is higher than that of SnS_2_/G (0.15 e). Figures 3h and S11b depict the band structures of A-SnS_2_@G and SnS_2_/G in detail. In case of A-SnS_2_@G, featured by some conduction bands downshift toward valence band, metallic electronic structure is constructed, which pushes to a reinforced electrical conductivity [40]. Moreover, the broadened bands for A-SnS_2_@G result in smaller electron effective mass, indicative of superior electron mobility under the external electric field. What's more, the calculated density of states (DOS) for A-SnS_2_@G around the Fermi level is much higher than that of SnS_2_/G with the common van der Waals interaction (Fig. 3i), further certificating the enhanced electron migration [9].

### Sodium Storage Performance

**Fig. 4:**
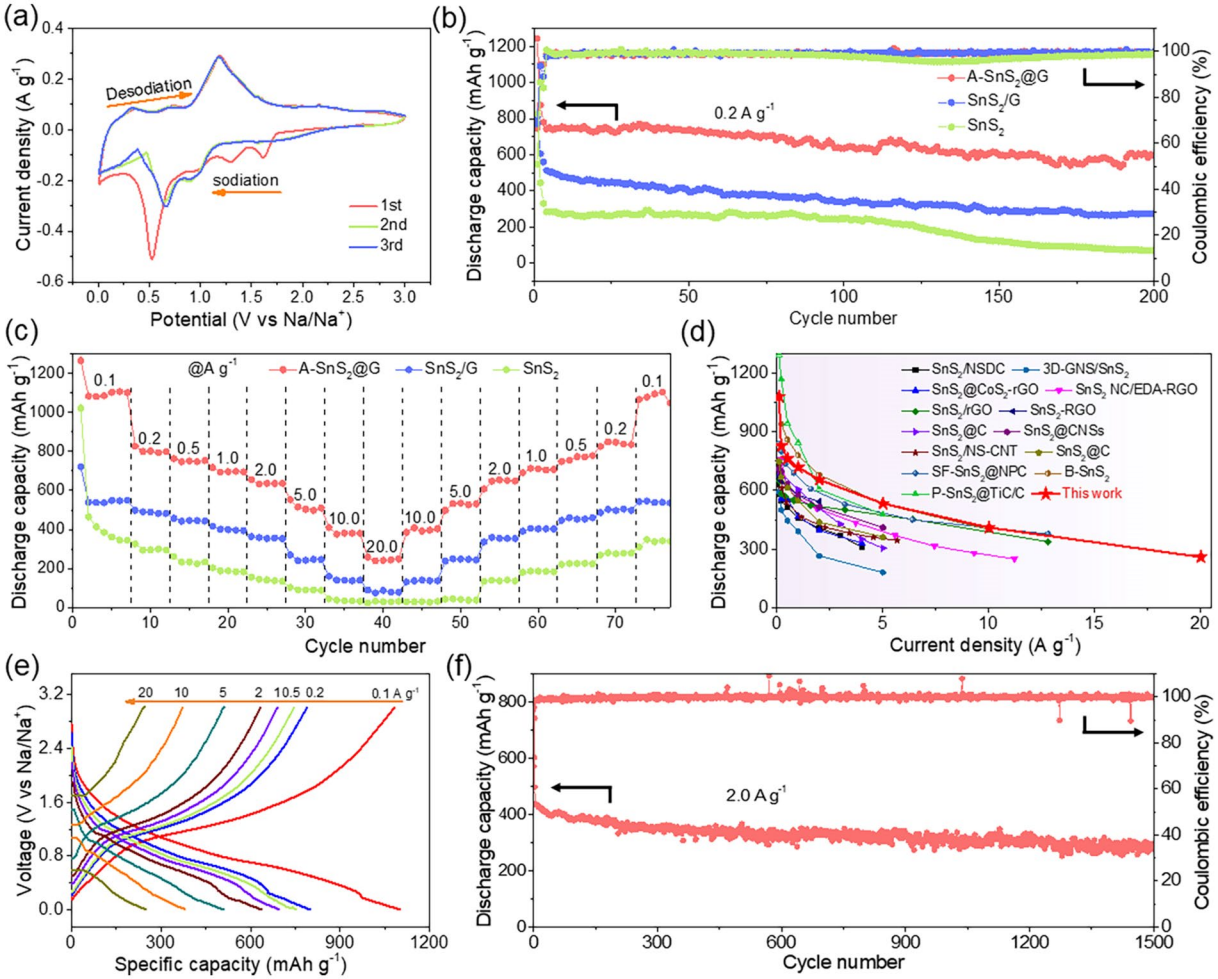
**a** CV curves of A-SnS_2_@G at a scan rate of 0.1 mV s^−1^. **b** Cycling performance at 0.2 A g^−1^ and **c** rate capability of A-SnS_2_@G, SnS_2_/G and SnS_2_. **d** Comparison of rate capability with previously reported SnS_2_-based anodes for SIBs. **e** Charge–discharge profiles of A-SnS_2_@G electrode at various sweep rates. **f** Long-term cycling performance of A-SnS_2_@G at 2.0 A g^−1^

Inspired by the covalent face-to-face bridging in the heterostructure of A-SnS_2_@G with metalloid electron properties that promises dramatic Na-storage kinetics, eminent cycling and rate capability, its application in Na storage was systematically investigated. Figure 4a displays the cyclic voltammetry (CV) curves of A-SnS_2_@G at a scan rate of 0.1 mV s^−1^. The peaks at high voltage (1.0, 1.3 and 1.6 V) in the first cathodic scan could be attributed to sodium intercalation into SnS_2_ layers. The distinct cathodic peak at 0.5 V ascribes to the Na-Sn alloying process as well as the formation of irreversible solid electrolyte interface (SEI) layer, which splits into two weak peaks at 0.65 and 0.09 V in subsequent cycles. In the anodic sweeps, the corresponding oxidation peaks (0.35, 0.71 and 1.18 V) may be assigned to the desodiation of Na_*x*_Sn as well as the restitution of SnS_2_ [15]. The voltammograms almost overlap in the following cycles, an indication of the excellent electrochemical reversibility upon Na storage. Galvanostatic discharge–charge (GDC) was performed to determine the Na-storage performance of A-SnS_2_@G, SnS_2_/G and SnS_2_ anodes (Fig. 4b). Obviously, the A-SnS_2_@G delivers far superior reversible capacity of 597 mAh g^−1^ at 0.2 A g^−1^ after 200 cycles with high average Coulombic efficiency (CE) close to 100%. The capacity is 2.2 and 8.4 times as high as that of SnS_2_/G (275 mAh g^−1^) and SnS_2_ (71 mAh g^−1^), highlighting the robustness and ample Na-storage sites of such heterostructure with covalent bridging.

Rate capability is an important index to reveal the ion-storage kinetics of electrode materials. As shown in Fig. 4c, A-SnS_2_@G delivers capacities of 1081, 827, 763, 718, 655, 553 and 410 mAh g^−1^ at stepwise current densities from 0.1 to 10.0 A g^−1^, respectively. Impressively, unprecedented rate capacity of 259 mAh g^−1^ is still achieved even at an extreme current rate of 20.0 A g^−1^, indicative of a super-fast Na-storage kinetics by high-flux electron/ion migration. Moreover, the reversible capacity is almost fully recovered (1066 mAh g^−1^) when the current density switches back to 0.1 A g^−1^, elucidating the excellent reversibility of A-SnS_2_@G. In sharp contrast, SnS_2_/G and SnS_2_ display much lower capacities than A-SnS_2_@G at various rates. To our knowledge, the rate capability of as-prepared A-SnS_2_@G shows prominent superiority when compared with recently reported SnS_2_-based anode for SIBs, especially at current density higher than 5.0 A g^−1^ (Fig. 4d and Table S3). Such a reinforcement on Na-storage performance could be originated from the high-flux electron/ion transfer boosted by the consecutive interfacial covalent bridging with face-to-face contact.

The discharge/charge curves of A-SnS_2_@G at different rates are displayed in Fig. 4e. The sodiation/desodiation plateaus are in good accordance with the CV curves, delivering low polarizations with the increase of current density. The initial discharge/charge process of A-SnS_2_@G at 0.1 A g^−1^ records high specific capacities of 1265 and 869 mAh g^−1^, leading to an initial CE of 68.7% (Fig. S12), higher than that of SnS_2_/G (68.5%) and SnS_2_ (42.8%). The irreversible capacity loss mainly stems from the formation of SEI film as well as the irreversible reaction with residual functional groups on the rGO [51–54]. Inspired by excellent rate capability, the long-term cycling of A-SnS_2_@G at 2.0 A g^−1^ is performed, as shown in Fig. 4f. Delightfully, a considerable capacity of 295 mAh g^−1^ still retains even after 1500 cycles at 2.0 A g^−1^.

**Fig. 5:**
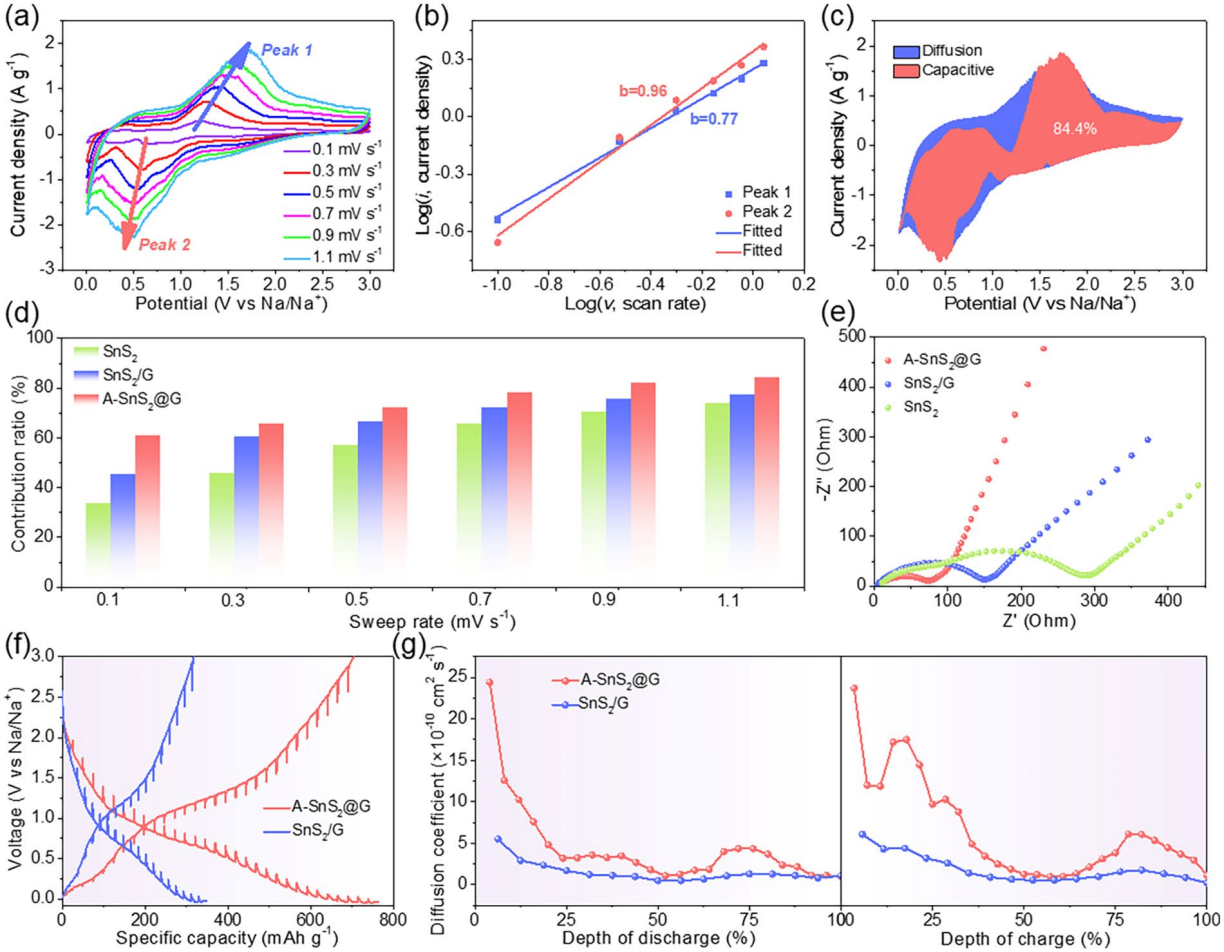
**a** CV curves of A-SnS_2_@G at 0.1–1.1 mV s^−1^. **b** Corresponding log(*i*) versus log(*v*) plots for anodic and cathodic peaks. **c** Capacitive contribution at 1.1 mV s^−1^ for A-SnS_2_@G. **d** Comparison of capacitive contribution for A-SnS_2_@G, SnS_2_/G and SnS_2_. **e** EIS for A-SnS_2_@G, SnS_2_/G and SnS_2_ in the 200 cycles at 0.2 mV s^−1^. **f** GITT curves and **g** corresponding Na^+^ diffusion coefficients of A-SnS_2_@G and SnS_2_/G electrodes at the 50th cycle

CV measurements at different scan rates were conducted to deeply investigate the contribution of covalent bridging on Na-storage kinetics (Figs. 5a, S13a and S14a). All the CV curves of A-SnS_2_@G electrode display resembling shapes, indicative of the superior electron/ion conductivity and low polarization. Generally, the contribution of diffusion or capacitance-limited process can be quantified via current (*i*) and sweep rate (*v*) according to the power-law equation (Eq. (2)):

2 $$\log \left( i \right) = b\log \left( v \right) \, + \, \log \left( a \right)$$

where *b*-value adjusts from 0.5 (diffusion-dominated behavior) to 1 (capacitive-controlled behavior) [55]. The calculated *b*-values of A-SnS_2_@G anode are closer to 1 than that of SnS_2_/G and SnS_2_ anodes (0.77 vs. 0.74 vs. 0.69 for the peak 1; 0.96 vs. 0.90 vs. 0.42 for the peak 2), demonstrating that the covalent bridging is favorable for the surface-induced pseudocapacitance response (Figs. 5b, S13b and S14b). The capacitive (*k*_1_*v*) and diffusion-dominated (*k*_2_*v*^1/2^) contribution can be quantitatively analyzed according to Eq. (3) [56, 57].

3 $$i\left( v \right) = k_{1} v + k_{2} v^{1/2}$$

As shown in Figs. 5c, S13c and S14c, the capacitive-dominated areas (dark pink) are highlighted in the CV curves at 1.1 mV s^−1^. A dominantly capacitive contribution of 84.4% is achieved for A-SnS_2_@G, significantly higher than 77.3% for SnS_2_/G and 73.8% for SnS_2_. The capacitive contributions from 0.1 to 1.1 mV s^−1^ are summarized in Fig. 5d by analysis of all other sweep rates (Figs. S13d and S14d). As expected, the A-SnS_2_@G electrode delivers the highest capacitive contribution at all sweep rates, answerable to the excellent Na-storage kinetics. EIS was collected to further investigate the facilitated charge transfer of A-SnS_2_@G (Fig. 5e). The kinetic parameters are well-fitted according to the equivalent circuit model (Fig. S15 and Table S2). In contrast, A-SnS_2_@G possesses the lowest *R*_*ct*_ value (57 Ω) compared with SnS_2_/G (141 Ω) and pure SnS_2_ (270 Ω), indicative of the accelerated electron/ion migration by the covalent bridging effect [58, 59]. The diffusion kinetics of Na^+^ in different charge/discharge depths were evaluated in details by galvanostatic intermittent titration technique (GITT) in a coin cell after 50 cycles. The Na^+^ diffusion coefficient (D_Na+_) can be calculated according to Fick's second law (Eq. (4)):

4 $$D = \frac{4}{\pi \tau }\left( {\frac{{m_{B} V_{M} }}{{M_{B} A}}} \right)^{2} \left( {\frac{{\Delta E_{s} }}{{\Delta E_{\tau } }}} \right)^{2}$$

where *τ* is the current pulse time, *m*_*B*_, *M*_*B*_ and *V*_*M*_ are the mass, molar mass and molar volume of the active material, *A* is the electrode–electrolyte interfacial area, ∆*E*_*s*_ and ∆*E*_*t*_ are the quasi-equilibrium potential and the change of cell voltage *E* during the constant current pulse, respectively [60]. Obviously, A-SnS_2_@G displays smaller overpotentials when comparing with SnS_2_/G, corroborating a higher D_Na+_ (Fig. 5f). The D_Na+_ as a function of the discharge/charge depth is shown in Fig. 5g. A-SnS_2_@G delivers much higher D_Na+_ than SnS_2_/G throughout the whole sodiation/desodiation process, especially at the Na^+^ insertion stage during discharge process and dealloying stage upon charging. The average D_Na+_ was calculated to be 5.5 × 10^−10^ cm^2^ s^−1^ for A-SnS_2_@G, 3.2 times as much as that of SnS_2_/G electrode (1.7 × 10^−10^ cm^2^ s^−1^).

**Fig. 6:**
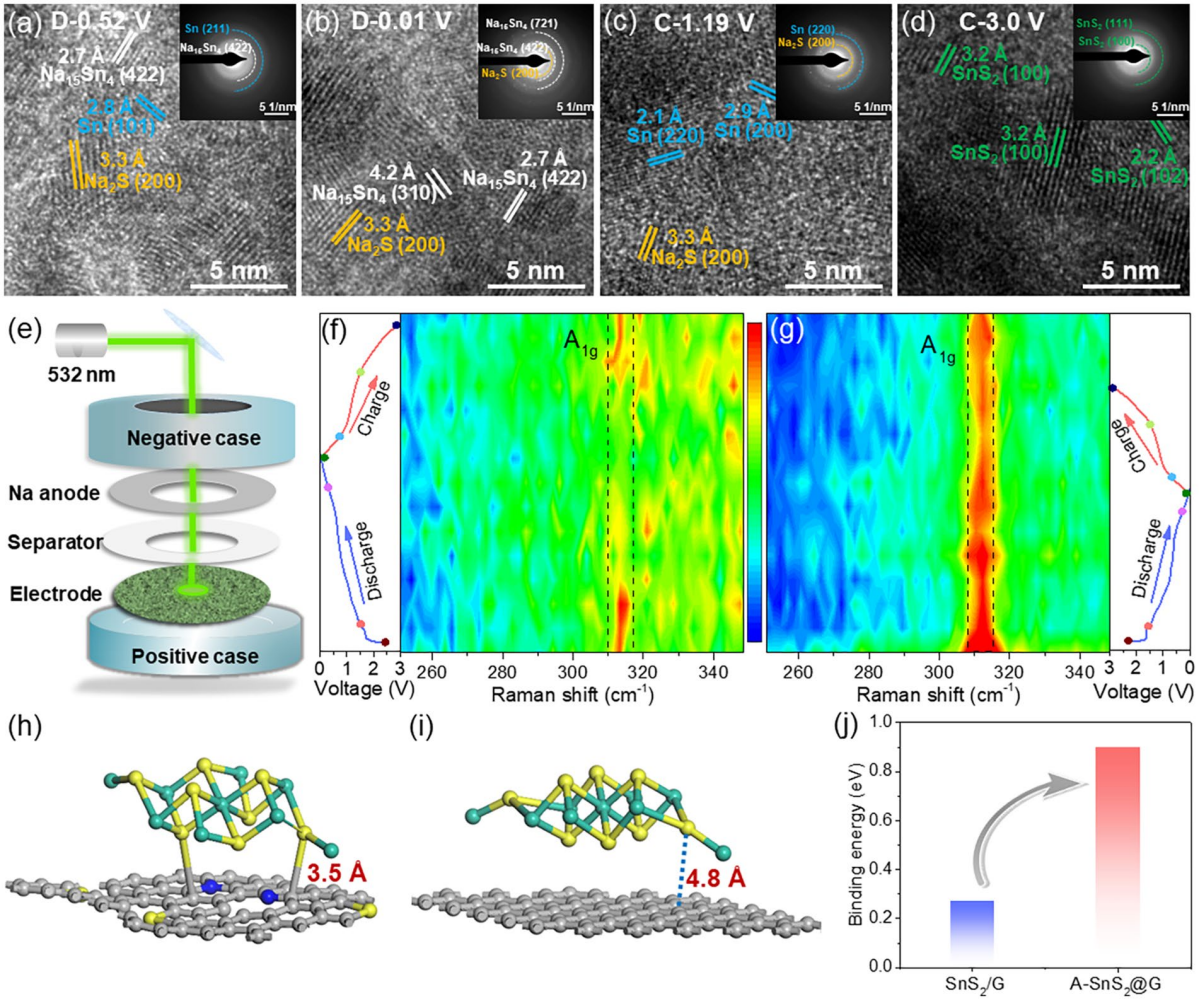
**a**–**d** Ex situ HRTEM images of A-SnS_2_@G electrode at varying charge/discharge state (the insets are corresponding SAED patterns). **e** Schematic illustration of in situ Raman measurement. In situ Raman spectra of **f** A-SnS_2_@G and **g** SnS_2_/G at various discharge–charge depths. Optimized structures of **h** A-SnS_2_@G and **i** SnS_2_/G, respectively. **j** Calculated binding energy of SnS_2_ on graphene for A-SnS_2_@G and SnS_2_/G

The variation of the electrode structures before and after cycling was supervised to verify the improved stability of A-SnS_2_@G electrode upon cycling. From the ex situ SEM images, A-SnS_2_@G electrode still keeps the structural wholeness and intimate adheres to Cu foil with a thickness of ~ 34 μm after 200 cycles (Fig. S16c, d) compared to fresh A-SnS_2_@G electrode (Fig. S16a-b). In striking contrast, severe cracks and breakaway from the Cu foil occur in SnS_2_/G electrode with a further inflated electrode thickness (~ 43 μm) after cycling (Fig. S16e, f). Ex situ TEM and SAED were performed to gain further insight into the phase evolution of A-SnS_2_@G electrode at various discharge/charge states. As shown in Fig. 6a, Sn and Na_9_Sn_4_ are observed except the conversion reaction (SnS_2_ + 4Na^+^  + 4e^−^  ↔ Sn + 2Na_2_S) product Na_2_S, indicating that partial alloying occurs when discharged to 0.52 V. When fully discharged to 0.01 V (Fig. 6b), the Sn undergoes complete sodiation to Na_15_Sn_4_ (4Sn + 15Na^+^  + 15e^−^  ↔ Na_15_Sn_4_). The HRTEM image in Fig. 6c reveals that the completion of dealloying reaction and Sn is separated out again after recharging back to 1.19 V. Upon fully charged to 3.0 V, SnS_2_ recovers to the original phase as demonstrated by the (100), (102) and (111) fringes in Fig. 6d and inserted SAED pattern, verifying the improved reversibility and ion-storage kinetics of A-SnS_2_@G electrode.

Furthermore, in situ Raman technology (Fig. 6e) was conducted under a sweep rate of 1.0 mV s^−1^ to confirm the high reversibility of A-SnS_2_@G electrode. As shown in Fig. 6f, the typical A_1g_ peak of the A-SnS_2_@G electrode gradually decays and fully disappears with gradually discharging to 0.01 V, indicative of the synchronous sodiation of SnS_2_ under rapid input current. The peak remerges after fully desodiation, further confirming the Na-storage reversibility of our proposed covalent-bridging heterostructure with face-to-face contact. In sharp contrast, the intensity of the A_1g_ peak enlarges and almost no changes upon sodiation/desodiation process for SnS_2_/G electrode (Fig. 6g), implying that numerous SnS_2_ cannot be sodiated even after fully charging, which is responsible for the sluggish Na-storage kinetics.

Quantitatively, the binding energies (*E*_*b*_) were calculated to investigate the adhesion level between SnS_2_ nanosheets and graphene matrix. The distance between C and S is ~ 3.5 Å for optimized A-SnS_2_@G heterostructure (Fig. 6h), much shortness compared with SnS_2_/G composite (4.8 Å) without C-S-Sn bonds (Fig. 6i). Consistently, the calculated *E*_*b*_ for A-SnS_2_@G is − 0.9 eV (Fig. 6j), 3.3 times as much as the *E*_*b*_ for SnS_2_/G (− 0.27 eV). The intimate adhesion of SnS_2_ to graphene matrix via face-to-face covalent bridging is beneficial to anchoring Sn and Na_x_Sn intermediates upon sodiation/desodiation, imparting a significant enhancement on long-term cycling stability.

To further evaluate the potential application of A-SnS_2_@G, a Na^+^ full cell was assembled with Na_3_V_2_(PO_4_)_3_ as cathode and A-SnS_2_@G as anode (Fig. S17a). The capacity ratio of cathode/anode was ~ 1.2:1 to ensure the optimized Na-storage capability. As depicted in Fig. S17b, the as-prepared full cell delivers a reversible capacity of 221 mAh g^−1^ at 0.5 A g^−1^ after 1000 cycles based on the mass of A-SnS_2_@G anode. Visually, the digital image implanted in Fig. S17b indicates that the as-assembled full cell can readily light a light-emitting diodes (LEDs) pattern, certification of a potential application of our proposed A-SnS_2_@G anode materials.

## Conclusions

In summary, this work demonstrates an efficient strategy of laser-derived interfacial confinement that could yield heterostructures of 2D-nanosheets/graphene with planar interfaces, by employing the prebonding of laser-manufactured metastable nanoparticles on graphene, as well as the amorphous feature of the nucleated nanoparticles that could drive the parallelly epitaxial growth of the anchored 2D nanosheets. The resulted face-to-face contact in as-prepared heterostructure enables huge covalent coupling area, unimpeded electron/ion transfer pathways and indestructible structural stability. Featured by the successive covalent bridging and ultrathin layer structure (~ 4 layers), the heterostructure was experimentally verified to boost the electron/ion kinetics, possess more metal-ions storage sites and guarantee structural integrity, thus resulting in extraordinary reversible capacity (597 mAh g^−1^ at 0.2 A g^−1^ over 200 cycles) and rate capability (259 mAh g^−1^ at 20.0 A g^−1^). The obtained Na-storage performance in present work ranks at top in the records of SnS_2_-based anodes for SIBs. The DFT simulations further clarify that the firm covalent bridging pledges the spontaneous electron/ion transfer in-between the heterointerface. We thus anticipate that our findings could pave new opportunities for addressing the inferior interfacial bridging in between the heterostructures via creating amorphous supranano particles in a myriad of applications based on laser–matter interactions.

## Supplementary Information

Below is the link to the electronic supplementary material.

Supplementary file1 (DOCX 5253 KB)
