# Supplementary material for: Laser-Derived Interfacial Confinement Enables Planar Growth of 2D SnS2 on Graphene for High-Flux Electron/Ion Bridging in Sodium Storage
Source: Nanomicro Lett. 2022 Apr 1;14:91. doi: 10.1007/s40820-022-00829-1 (PMC8975989; doi:10.1007/s40820-022-00829-1)
Supplement: Supplementary file 1 — Supplementary file1 (DOCX 5253 KB) [file 40820_2022_829_MOESM1_ESM.docx]

Supporting Information for

**Laser Derived Interfacial Confinement Enables Planar Growth of 2D SnS_2_ on Graphene for** **High-Flux Electron/Ion Bridging in** **Sodium** **Storage**

Xiaosa Xu^#^, Fei Xu^#^, Xiuhai Zhang, Changzhen Qu, Jinbo Zhang, Yuqian Qiu, Rong Zhuang, and Hongqiang Wang*

State Key Laboratory of Solidification Processing, Centre for Nano Energy Materials, School of Materials Science and Engineering, Northwestern Polytechnical University, Shaanxi Joint Laboratory of Graphene (NPU), Xi’an, 710072, P. R. China.

^#^ These authors contributed equally to this work.
*Corresponding author. E-mail: hongqiang.wang@nwpu.edu.cn (Hongqiang Wang)

**S1 Theoretical Calculation**

DFT calculations were conducted via CASTEP program along with the generalized gradient approximation (GGA) from the Perdew-Burke-Ernzerhof (PBE) defined electronic exchange-correlation interaction. The model systems were optimized based on the (2 × 2 × 1) SnS_2_ supercell on (6 × 6 × 1) graphene supercell. The vacuum distance of graphene was set as 15 Å to avoid interactions between neighboring layers. A cutoff energy of 517 eV was set and the Monkhorst-Pack k-point grid was 2×2×1. The convergence conditions for geometry optimization were as below: 1.0×10^−5^ eV/atom for energy, 0.03 eV/Å for force, 0.05 GPa for stress, and 0.001 Å for displacement, respectively. The Bader charge was obtained via analyzing the charge density gained from the VASP.

**S2 Supplementary Figures**

**
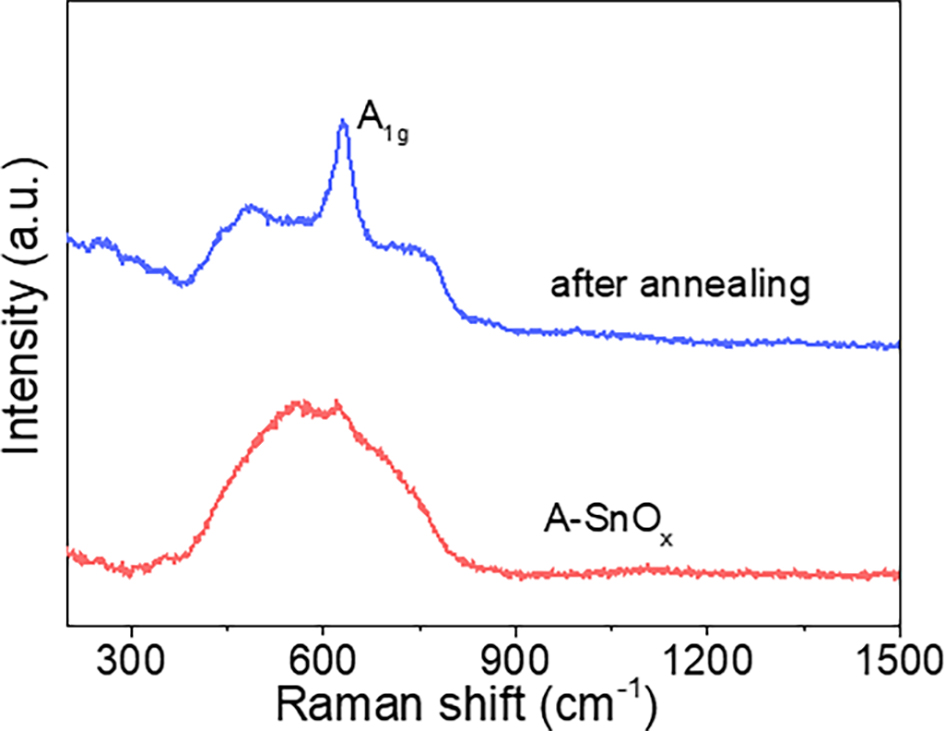
**

**Fig. S1** Raman patterns of A-SnO*_x_* and annealing in nitrogen


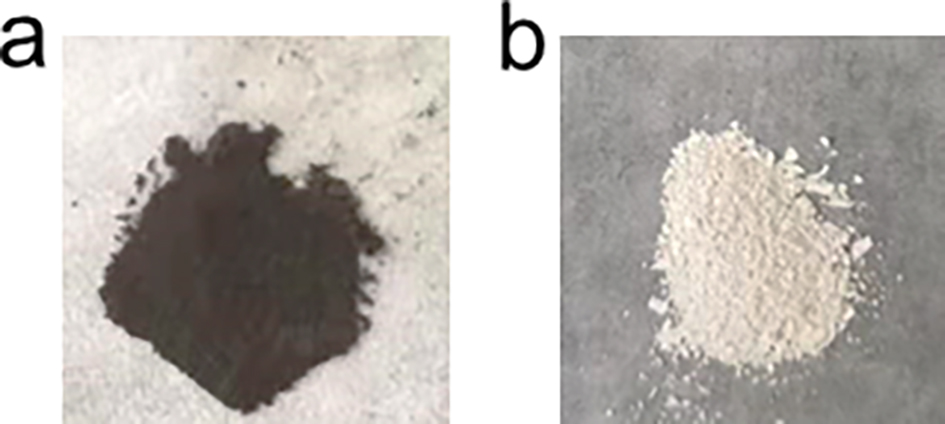


**Fig. S2** Photographs of **a** A-SnO*_x_* powder and **b** SnO_2_ powder


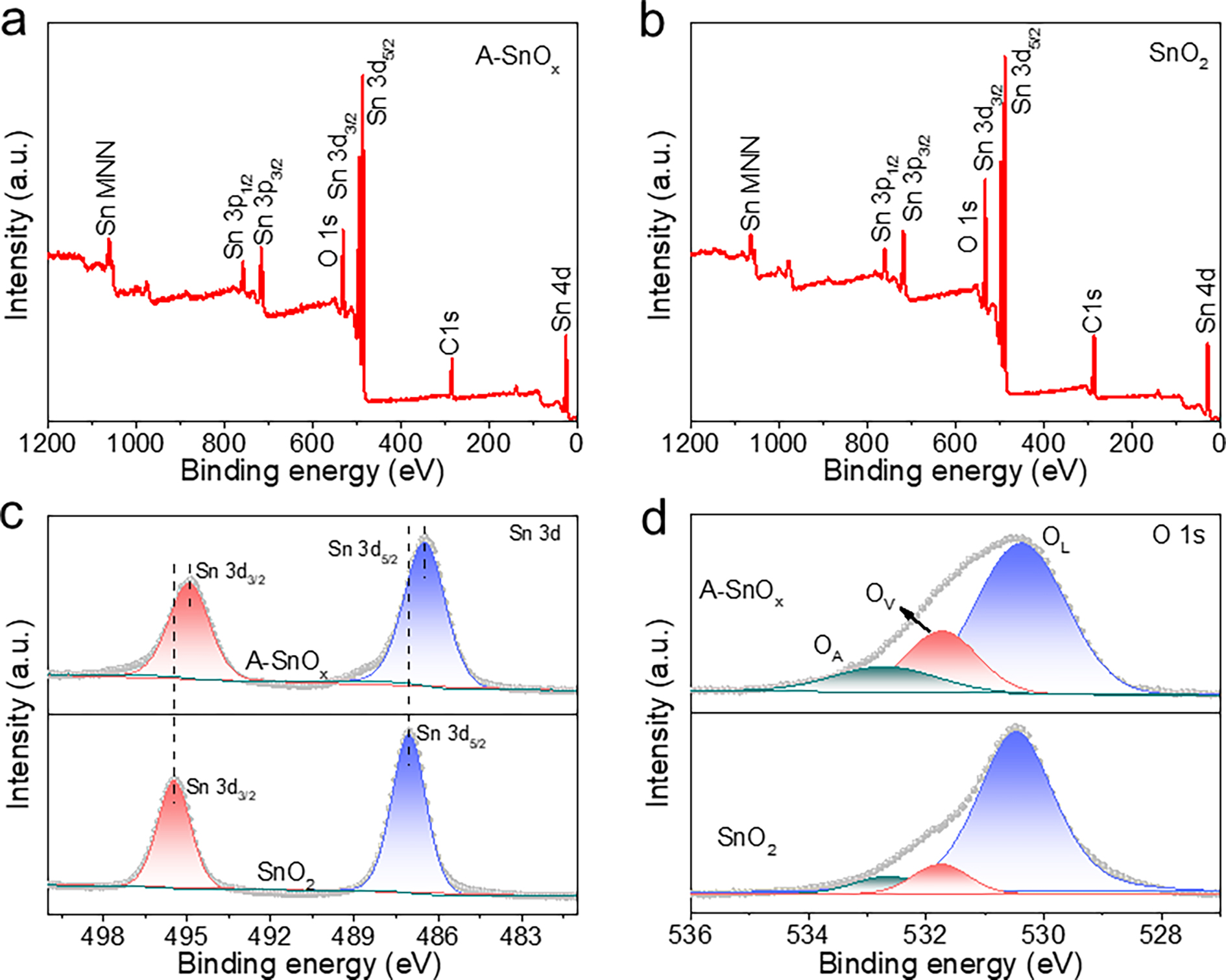


**Fig. S3** XPS survey spectra of **a** A-SnO*_x_* and **b** bulk SnO_2_. **c** XPS Sn 3d spectra and **d** O 1s spectra of A-SnO*_x_* and bulk SnO_2_

As shown in Fig. S3d, the fitted peak area assigned to oxygen vacancies in A-SnO*_x_* is much large than that of bulk SnO_2_. Combined with the obvious downshift of Sn 3d_5/2_ and 3d_3/2_ peaks of A-SnO*_x_* (Fig. S3c), it proves the existence of abundant oxygen vacancies in laser- manufactured A-SnO*_x_*.

**
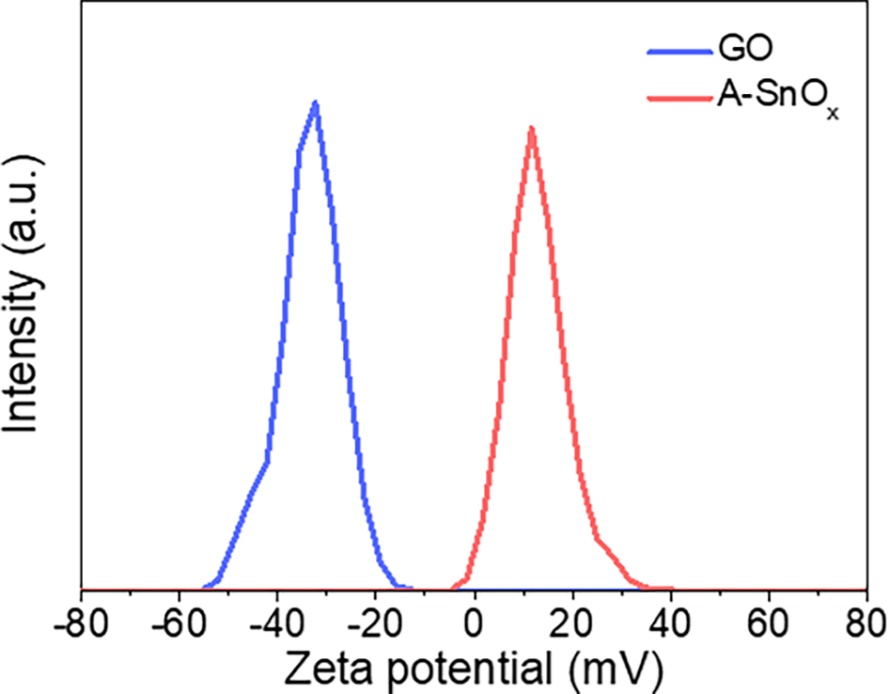
**

**Fig. S4** Zeta potentials of GO and A-SnO*_x_*

**
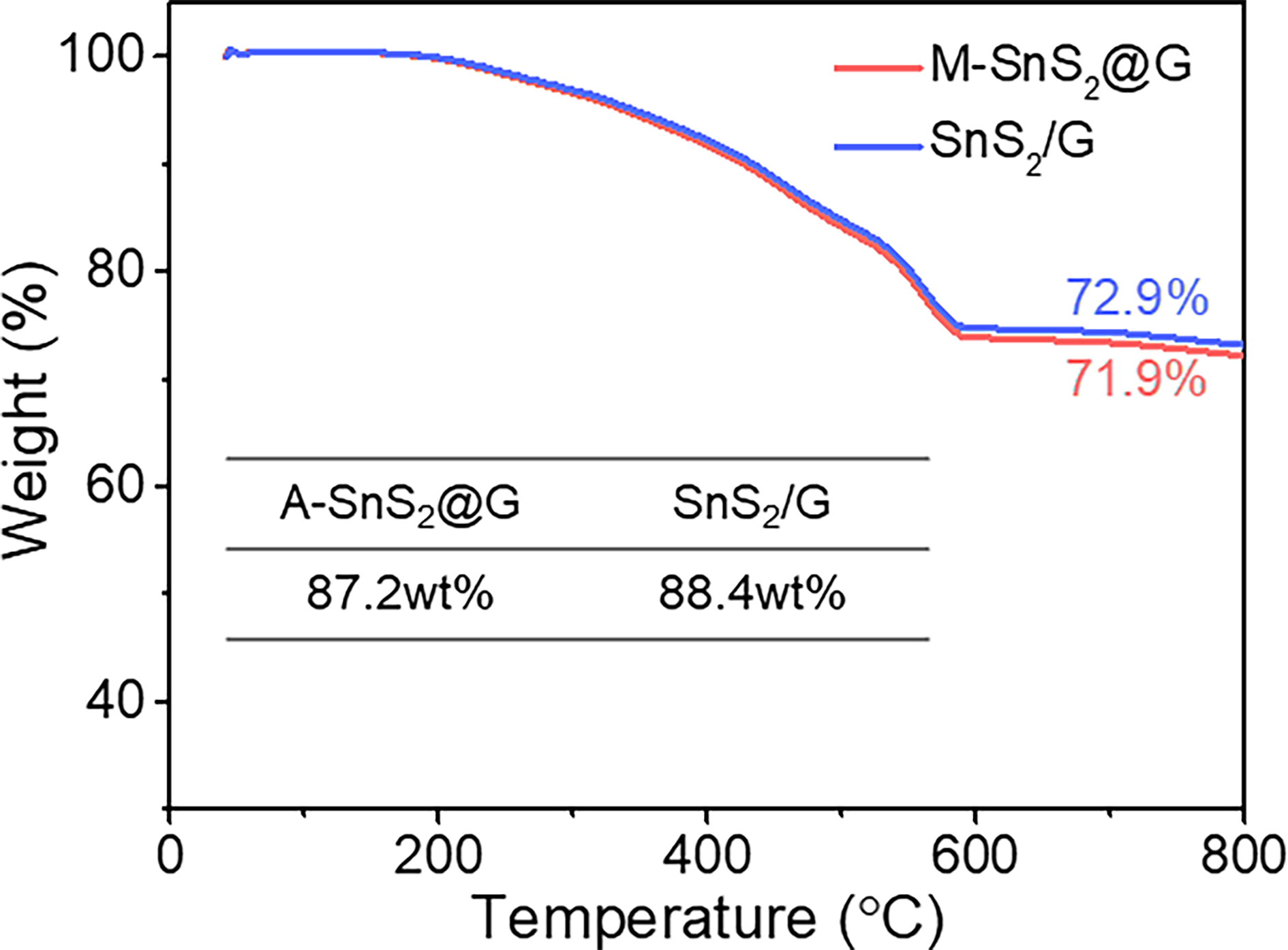
**

**Fig. S5** TGA curves of A-SnS_2_@G and SnS_2_/G


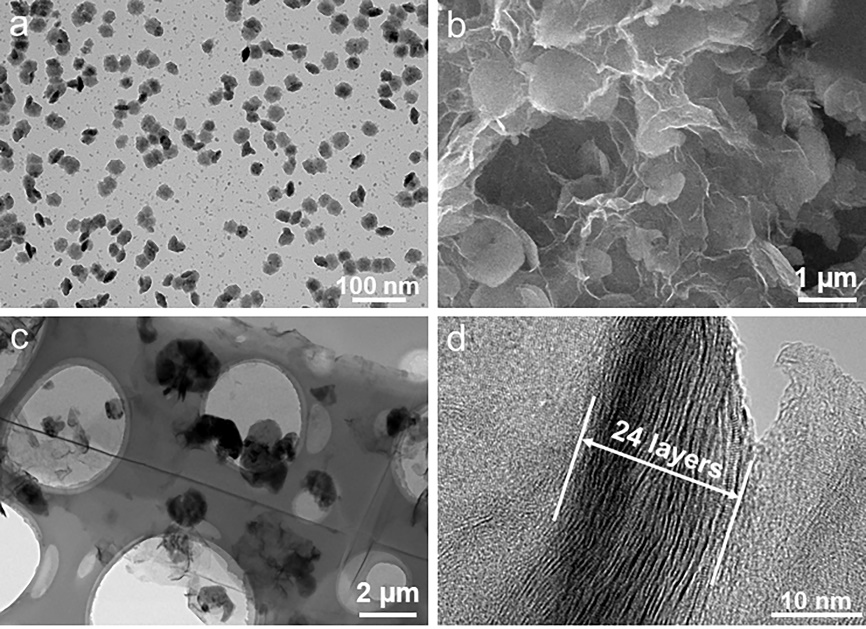


**Fig. S6 a** TEM image of large A-SnO*_x_* particles (~25 nm). **b** SEM image, **c** TEM image at low magnification, and **d** TEM image at high resolution of A-SnS_2_@G with large A-SnO*_x_* particles as seeds

**
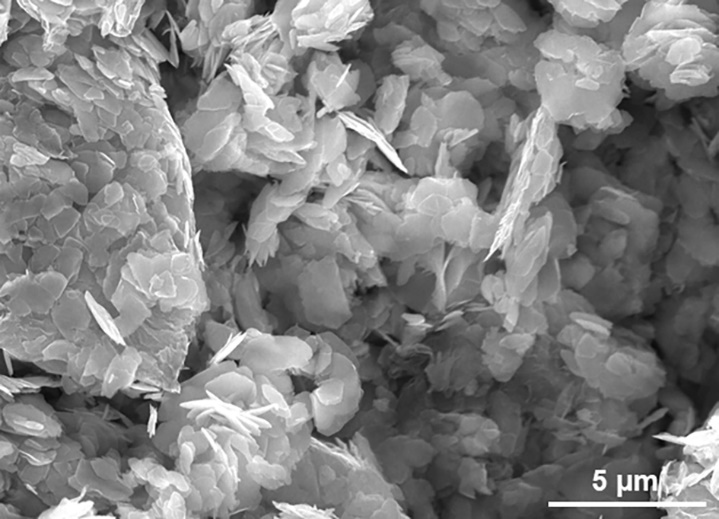
**

**Fig. S7** SEM image of SnS_2_


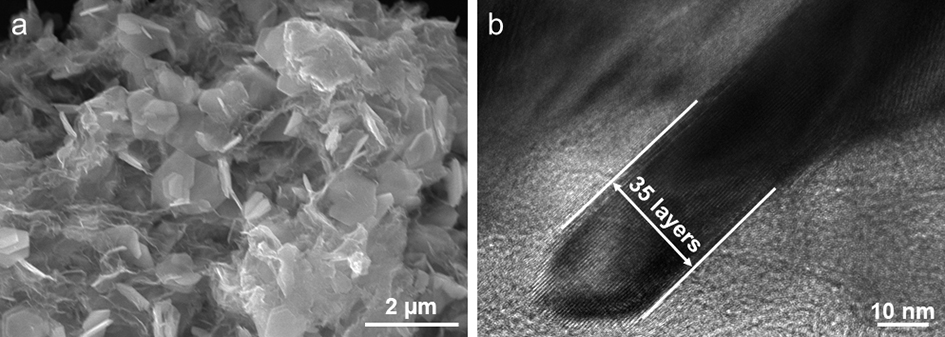


**Fig. S8 a** SEM and **b** TEM images of SnS_2_/G


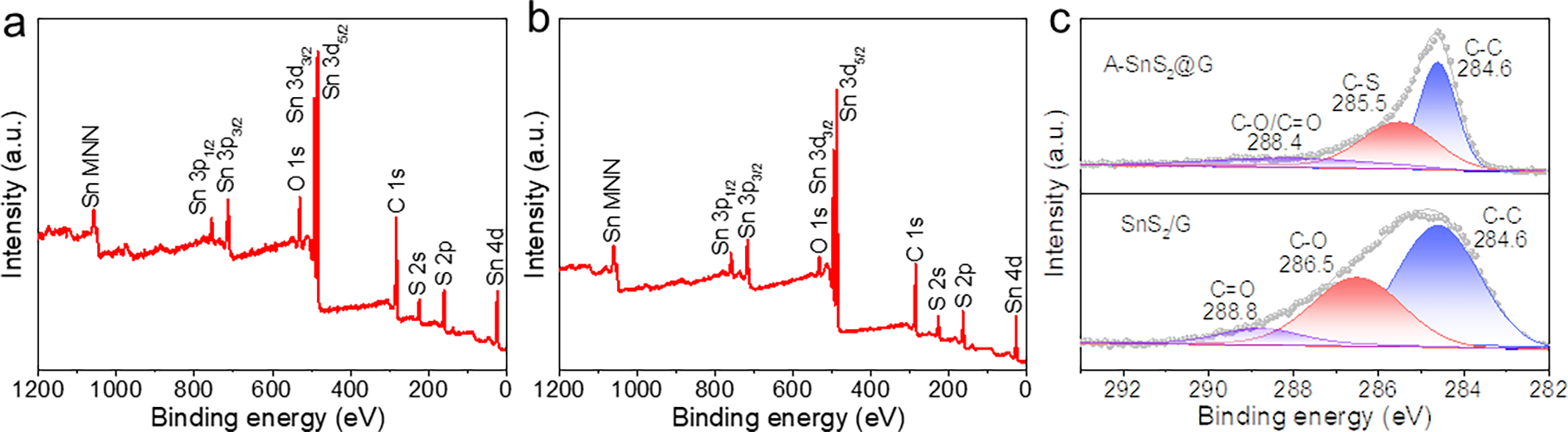


**Fig. S9** XPS survey spectra of **a** A-SnS_2_@G and **b** SnS_2_/G. **c** XPS C 1s spectra of A-SnS_2_@G and SnS_2_/G

**
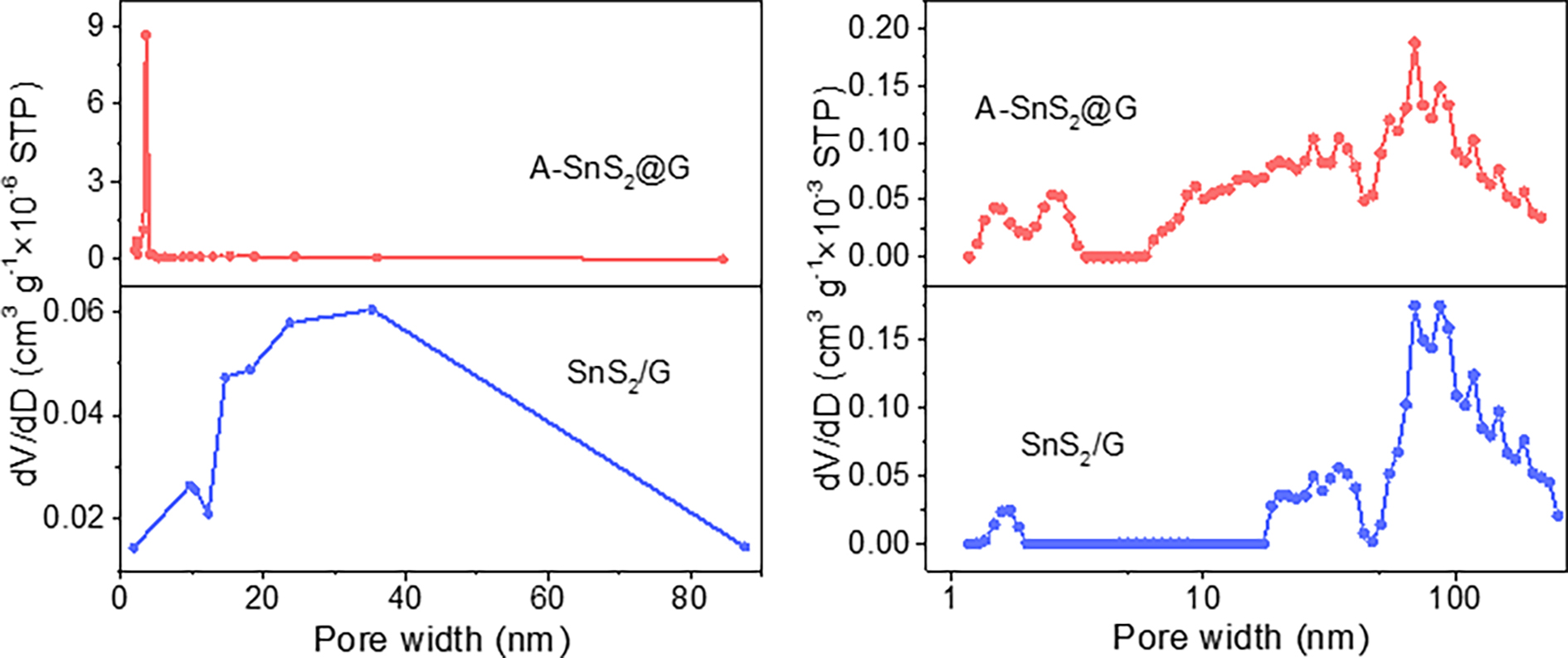
**

**Fig. S10** Pore size distribution curves of A-SnS_2_@G and SnS_2_/G

**
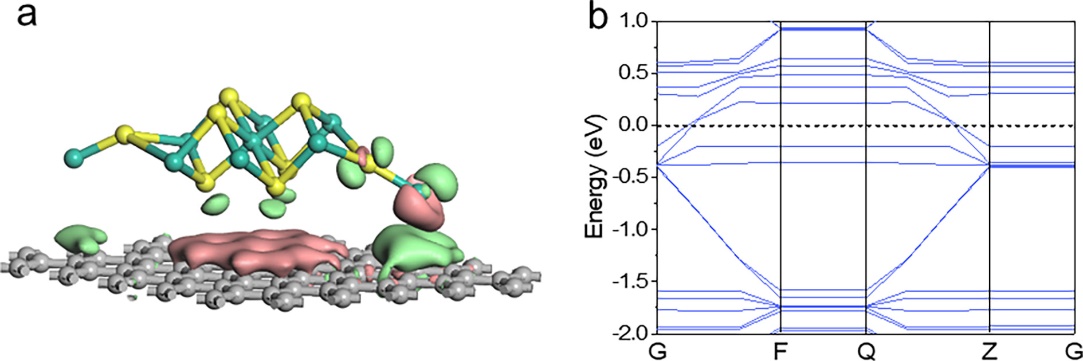
**

**Fig. S11 a** Charge density difference and **b** band structure of optimized A-SnS_2_@G model (the green/pink cloud represents the accumulation/depletion of electrons)

**
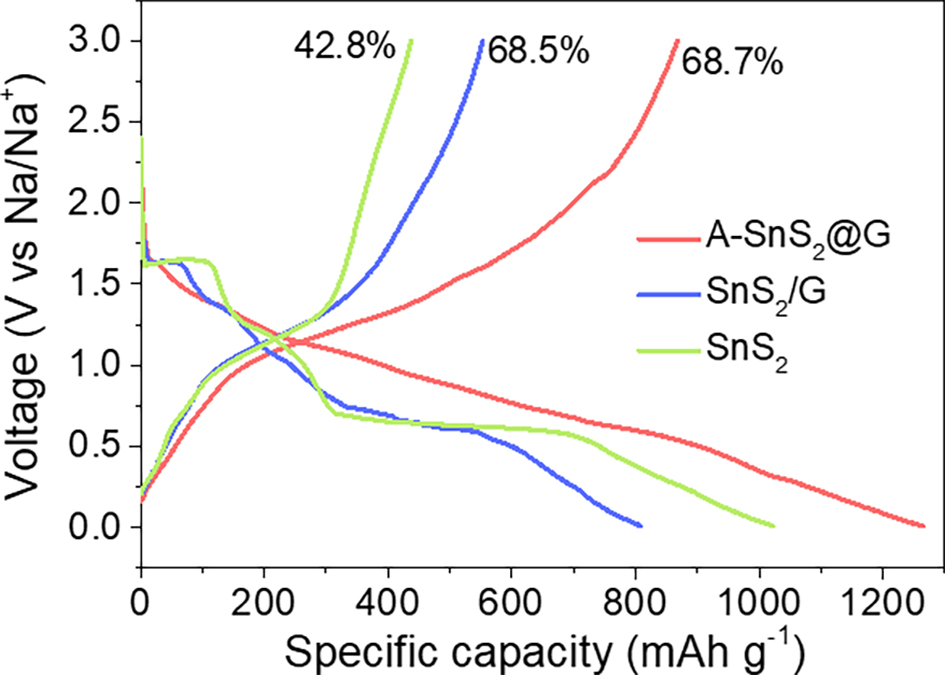
**

**Fig. S12** The initial charge/discharge profile of A-SnS_2_@G, SnS_2_/G, and SnS_2_ electrodes at 0.1 A g^-1^


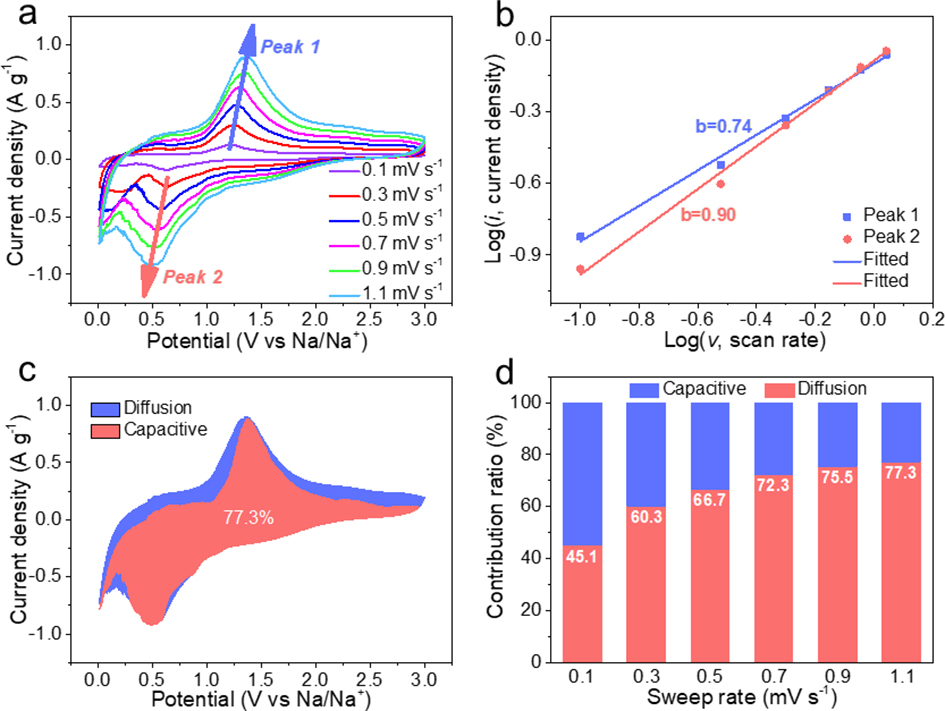


**Fig. S13** **a** CV curves of SnS_2_/G electrode at 0.1-1.1 mV s^-1^. **b** Corresponding log(*i*) versus log(*v*) plots for anodic and cathodic peaks. **c** Capacitive contribution at 1.1 mV s^-1^ for SnS_2_/G. **d** Contribution ratio of the capacitive and diffusion-limited capacity at different sweep rates


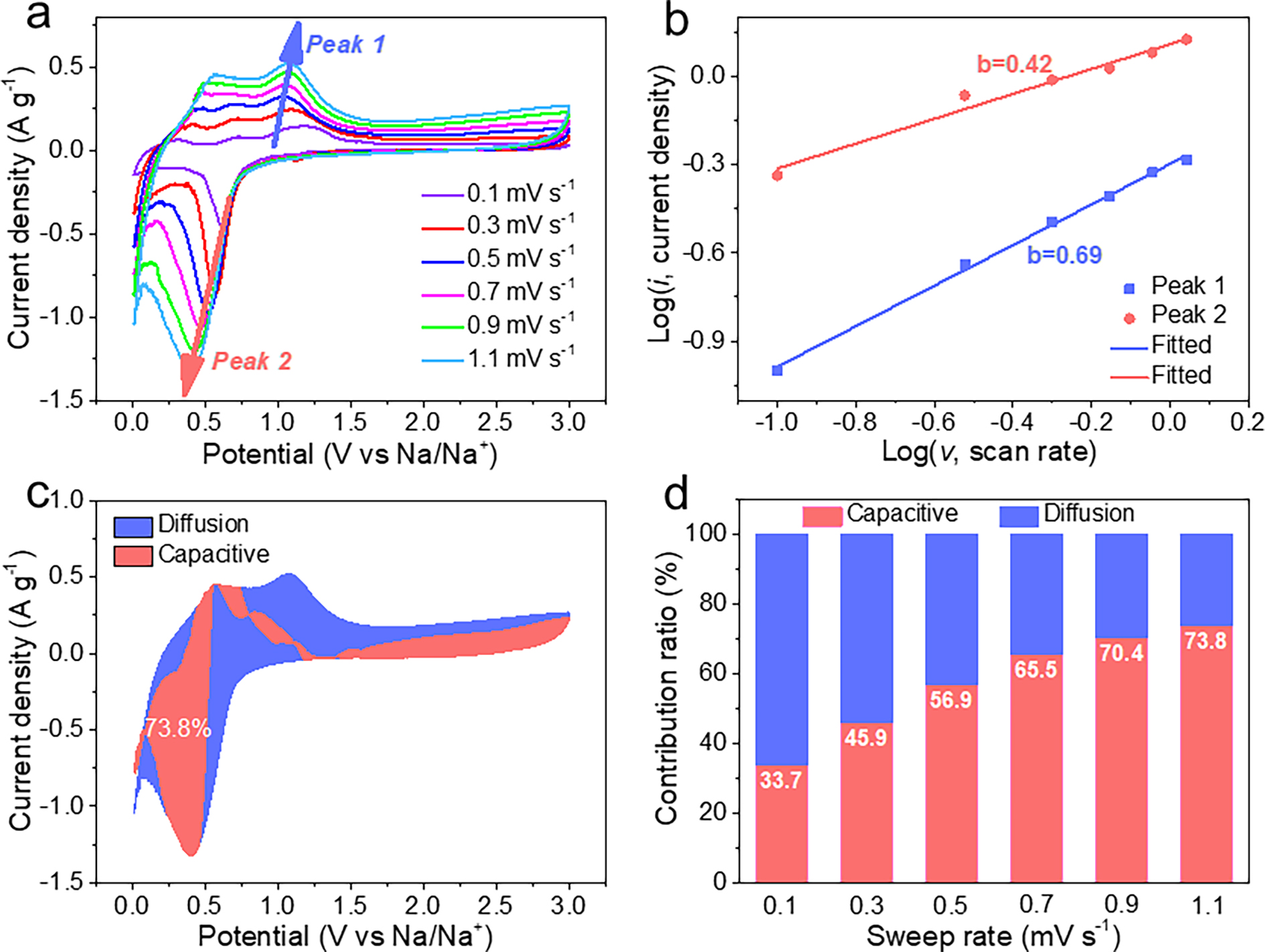


**Fig. S14** **a** CV curves of SnS_2_ electrode at 0.1-1.1 mV s^-1^. **b** Corresponding log(*i*) versus log(*v*) plots for anodic and cathodic peaks. **c** Capacitive contribution at 1.1 mV s^-1^ for SnS_2_. **d** Contribution ratio of the capacitive and diffusion-limited capacity at different sweep rates


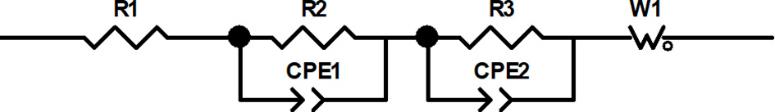


**Fig. S15** Corresponding equivalent circuit used to simulate EIS curves


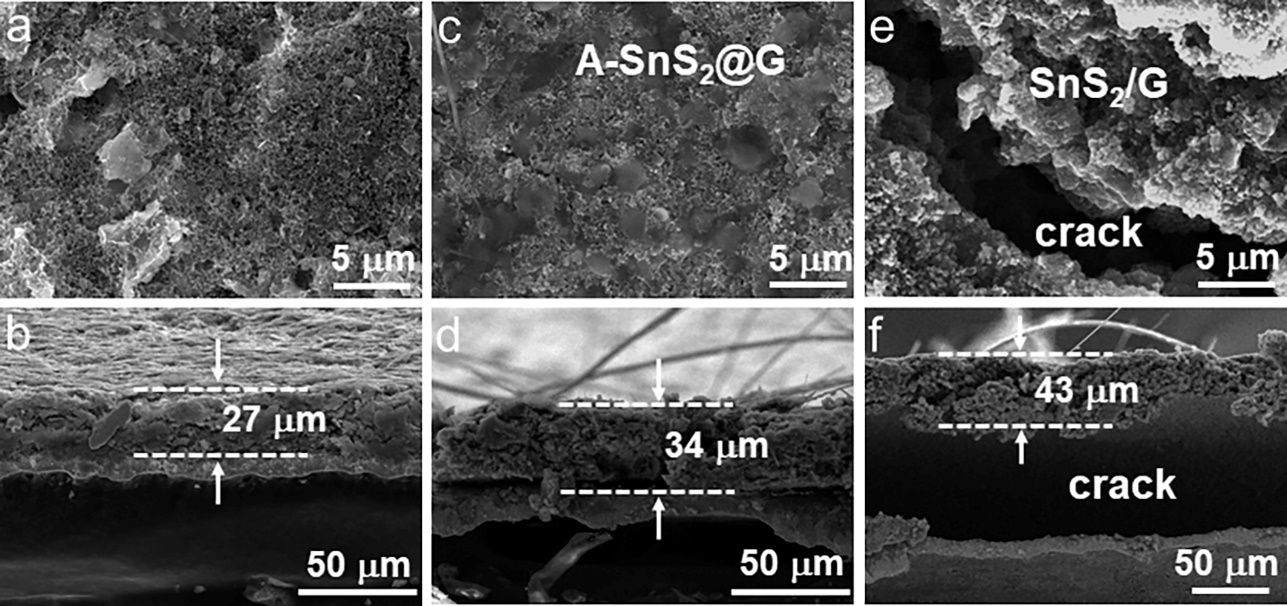


**Fig. S16** Top-view and cross-section SEM images of **a, b** fresh A-SnS_2_@G electrode, **c, d** A-SnS_2_@G electrode after cycling, and **e, f** SnS_2_/G electrode after cycling, respectively


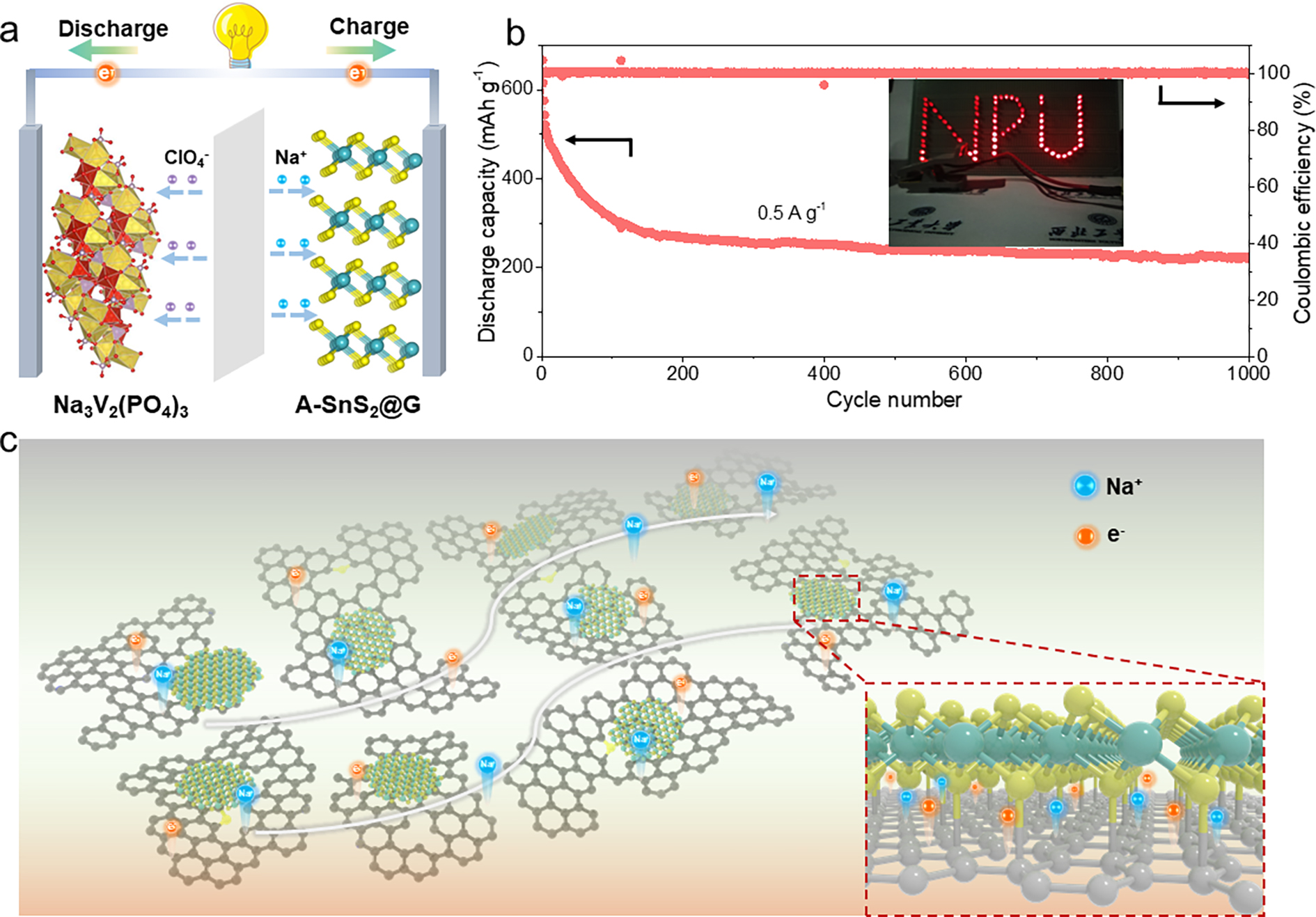


**Fig. S17** **a** Schematic illustration of the full cell configuration coupled with the Na_3_V_2_(PO_4_)_3_ cathode. **b** Cycling performance of the full cell at 0.5 A g^-1^ (the inset is digital image of LEDs lighted by a full cell). **c** Schematic illustration of interface bridging effect on Na-storage

**S3 Supplementary Tables**

**Table S1** Summary of the pore parameters for A-SnS_2_@G and SnS_2_/G.

| **Sample** | **S_BET_ (m^2^ g^-1^)** | **P_mic_ (%)** | **P_mes_ (%)** | **P_mac_ (%)** |
| --- | --- | --- | --- | --- |
| **A-SnS_2_@G** | 53.3 | 4.9 | 51 | 44.1 |
| **SnS_2_/G** | 19.6 | 3.0 | 18.7 | 78.3 |

Micro-, meso- and macropore ratios (P_mic_, P_mes_ and P_mac_, respectively) are calculated according to the following equations: P_mic_ = (V_mic_/V_sum_) x 100%, P_mes_ = (V_mes_/V_sum_) x 100%, and P_mac_ = 100% − P_mic_ − P_mes_, where V_mic_, V_mes_, V_mac_ and V_sum_ are the cumulative volume of Micro-, meso-, macro-pore and total pore.

**Table S2** The corresponding parameters from the equivalent circuit simulation.

| **Sample** | **R1 (Ohm)** | **R2 (Ohm)** | **R3 (Ohm)** | **CPE1 (F)** | **CPE2 (F)** |
| --- | --- | --- | --- | --- | --- |
| **A-SnS_2_@G** | 5.80 | 8.81 | 47.78 | 8.59E-06 | 3.51E-05 |
| **SnS_2_/G** | 4.06 | 9.49 | 131.9 | 1.04E-05 | 2.97E-05 |
| **SnS_2_** | 7.62 | 83.21 | 186.9 | 1.21E-05 | 4.87E-05 |

R_ct_=R2+R3.

**Table S3** Comparison of Na-storage performance of SnS_2_@NSG with the reported SnS_2_-based anode materials.

| Materials | Current density (A g^-1^) | | | | | | | | Ref. |
| --- | --- | --- | --- | --- | --- | --- | --- | --- | --- |
|  | 0.1 | 0.2 | 0.5 | 1 | 2 | 5 | 10 | 20 |  |
| SnS_2_/NSDC | 581.7 | 548.7 | 513.7 | 458.2 | 407.4 | -- | -- | -- | [S1] |
| 3D-GNS/SnS_2_ | 590 | 500 | 445 | 390 | 265 | 180 | -- | -- | [S2] |
| SnS_2_@CoS_2_-rGO | -- | 558 | 552 | 468 | 396 | -- | -- | -- | [S3] |
| SnS_2_ NC/EDA-RGO | 0.46 | 0.93 | 1.86 | 3.71 | 5.57 | 7.43 | 9.3 | 11.2 | [S4] |
|  | 630 | 560 | 510 | 435 | 370 | 315 | 280 | 250 |  |
| SnS_2_/rGO | 649 | 582 | 0.4 | 0.8 | 1.6 | 3.2 | 6.4 | 12.8 | [S5] |
|  |  |  | 570 | 550 | 524 | 501 | 452 | 337 |  |
| SnS_2_-RGO | 670 | 650 | 620 | 575 | 544 | -- | -- | -- | [S6] |
| SnS_2_@C | 695.5 | -- | -- | 604.1 | 507.6 | 304.4 | -- | -- | [S7] |
| SnS_2_@CNSs | 709 | 696 | 632 | 576 | 517 | 410 | -- | -- | [S8] |
| SnS_2_/NS-CNT | 0.11 | 0.23 | 0.45 | 1.14 | 2.27 | 3.41 | 4.54 | 5.68 | [S9] |
|  | 738 | 613 | 538 | 463 | 411 | 382 | 360 | 344 |  |
| SnS_2_@C | 750 | 668 | 614 | 548 | 438 | 362 | 452 | 337 | [S10] |
| SF-SnS_2_@NPC | 840 | 800 | 0.4 | 0.8 | 1.6 | 3.2 | 6.4 | 12.8 | [S11] |
|  |  |  | 735 | 690 | 608 | 530 | 450 | 378 |  |
| B-SnS_2_ | -- | 940 | 860 | 780 | 680 | 530 | 400 | -- | [S12] |
| P-SnS_2_@TiC/C | 1293.8 | 1169.8 | 943.3 | 843.7 | 605.8 | 476.4 | -- | -- | [S13] |
| SnS_2_/CNTs | 690 | 554 | 437 | 368 | 282 | -- | -- | -- | [S14] |
| **A-SnS_2_@G** | **1081** | **827** | **763** | **718** | **655** | **533** | **410** | **259** | **This work** |

Specific capacity: mAh g^-1^

**Supplementary References**

[S1] J. Xia, K. Z. Jiang, J. J. Xie, S. H. Guo, L. Liu et al., Tin disulfide embedded in N-, S-doped carbon nanofibers as anode material for sodium-ion batteries. Chem. Eng. J. **359**, 1244-1251 (2019). https://doi.org/10.1016/j.cej.2018.11.053

[S2] Z. Y. Sang, X. Yan, D. Su, H. M. Ji, S. H. Wang et al., A flexible film with SnS_2_ nanoparticles chemically anchored on 3D-graphene framework for high areal density and high rate sodium storage. Small **16**(25), 2001265 (2020). https://doi.org/10.1002/smll.202001265

[S3] X. Wang, X. Y. Li, Q. Li, H. S. Li, J. Xu et al., Improved electrochemical performance based on nanostructured SnS_2_@CoS_2_-rGO composite anode for sodium-ion batteries. Nano-Micro Lett. **10**(3), 46 (2018). https://doi.org/10.1007/s40820-018-0200-x

[S4] Y. Jiang, M. Wei, J. K. Feng, Y. C. Ma, S. L. Xiong, Enhancing the cycling stability of Na-ion batteries by bonding SnS_2_ ultrafine nanocrystals on amino-functionalized graphene hybrid nanosheets. Energy Environ. Sci. **9**(4), 1430-1438 (2016). https://doi.org/10.1039/c5ee03262h

[S5] Y. D. Zhang, P. Y. Zhu, L. L. Huang, J. Xie, S. C. Zhang et al., Few-layered SnS_2_ on few-layered reduced graphene oxide as Na-ion battery anode with ultralong cycle life and superior rate capability. Adv. Funct. Mater. **25**(3), 481-489 (2015). https://doi.org/10.1002/adfm.201402833

[S6] B. H. Qu, C. Z. Ma, G. Ji, C. H. Xu, J. Xu et al., Layered SnS_2_-reduced graphene oxide composite-a high-capacity, high-rate, and long-cycle life sodium-ion battery anode material. Adv. Mater. **26**(23), 3854-3859 (2014). https://doi.org/10.1002/adma.201306314

[S7] S. H. Li, Z. P. Zhao, C. Q. Li, Z. Y. Liu, D. Li, SnS_2_@C hollow nanospheres with robust structural stability as high-performance anodes for sodium ion batteries. Nano-Micro Lett. **11**(1), 14 (2019). https://doi.org/10.1007/s40820-019-0243-7

[S8] Y. Liu, X. Y. Yu, Y. J. Fang, X. S. Zhu, J. C. Bao et al., Confining SnS_2_ ultrathin nanosheets in hollow carbon nanostructures for efficient capacitive sodium storage. Joule **2**(4), 725-735 (2018). https://doi.org/10.1016/j.joule.2018.01.004

[S9] Z. J. Liu, A. Daali, G. L. Xu, M. H. Zhuang, X. B. Zuo et al., Highly reversible sodiation/desodiation from a carbon-sandwiched SnS_2_ nanosheet anode for sodium ion batteries. Nano Lett. **20**(5), 3844-3851 (2020). https://doi.org/10.1021/acs.nanolett.0c00964

[S10] Q. Sun, D. P. Li, L. N. Dai, Z. Liang, L. J. Ci, Structural engineering of SnS_2_ encapsulated in carbon nanoboxes for high-performance sodium/potassium-ion batteries anodes. Small **16**(45), 2005023 (2020). https://doi.org/10.1002/smll.202005023

[S11] X. Xu, R. S. Zhao, B. Chen, L. S. Wu, C. J. Zou et al., Progressively exposing active facets of 2D nanosheets toward enhanced pseudocapacitive response and high-rate sodium storage. Adv. Mater. **31**(17), 1900526 (2019). https://doi.org/10.1002/adma.201900526

[S12] D. L. Chao, P. Liang, Z. Chen, L. Y. Bai, H. Shen et al., Pseudocapacitive Na-ion storage boosts high rate and areal capacity of self-branched 2D layered metal chalcogenide nanoarrays. ACS Nano **10**(11), 10211-10219 (2016). https://doi.org/10.1021/acsnano.6b05566

[S13] Y. B. Shen, S. J. Deng, P. Liu, Y. Zhang, Y. H. Li et al., Anchoring SnS_2_ on TiC/C backbone to promote sodium ion storage by phosphate ion doping. Small **16**(40), 2004072 (2020). https://doi.org/10.1002/smll.202004072

[S14] L. Zhu, X. X. Yang, Y. H. Xiang, P. Kong, X. W. Wu, Neurons-system-like structured SnS_2_/CNTs composite for high-performance sodium-ion battery anode. Rare Metals **40**(6), 1383-1390 (2020). https://doi.org/10.1007/s12598-020-01555-6
